# A manager’s guide to using eDNA metabarcoding in marine ecosystems

**DOI:** 10.7717/peerj.14071

**Published:** 2022-11-15

**Authors:** Zachary Gold, Adam R. Wall, Teia M. Schweizer, N. Dean Pentcheff, Emily E. Curd, Paul H. Barber, Rachel S. Meyer, Robert Wayne, Kevin Stolzenbach, Kat Prickett, Justin Luedy, Regina Wetzer

**Affiliations:** 1 Department of Ecology and Evolutionary Biology, University of California, Los Angeles, Los Angeles, CA, United States of America; 2 Diversity Initiative for the Southern California Ocean (DISCO), Natural History Museum of Los Angeles County, Los Angeles, CA, United States of America; 3 Department of Fish and Wildlife Conservation Biology, Colorado State University, Fort Collins, CO, United States of America; 4 Department of Natural Sciences, Landmark College, Putney, VT, United States of America; 5 Department of Ecology and Evolutionary Biology, University of California, Santa Cruz, Santa Cruz, CA, United States of America; 6 Wood Environment and Infrastructure, Inc., San Diego, CA, United States of America; 7 Port of Los Angeles, Los Angeles, CA, United States of America; 8 Port of Long Beach, Long Beach, CA, United States of America

**Keywords:** eDNA, Metabarcoding, Management, Biomonitoring, Assessment, Biodiversity, Marine, Protocol, Primer set, Site occupancy

## Abstract

Environmental DNA (eDNA) metabarcoding is a powerful tool that can enhance marine ecosystem/biodiversity monitoring programs. Here we outline five important steps managers and researchers should consider when developing eDNA monitoring program: (1) select genes and primers to target taxa; (2) assemble or develop comprehensive barcode reference databases; (3) apply rigorous site occupancy based decontamination pipelines; (4) conduct pilot studies to define spatial and temporal variance of eDNA; and (5) archive samples, extracts, and raw sequence data. We demonstrate the importance of each of these considerations using a case study of eDNA metabarcoding in the Ports of Los Angeles and Long Beach. eDNA metabarcoding approaches detected 94.1% (16/17) of species observed in paired trawl surveys while identifying an additional 55 native fishes, providing more comprehensive biodiversity inventories. Rigorous benchmarking of eDNA metabarcoding results improved ecological interpretation and confidence in species detections while providing archived genetic resources for future analyses. Well designed and validated eDNA metabarcoding approaches are ideally suited for biomonitoring applications that rely on the detection of species, including mapping invasive species fronts and endangered species habitats as well as tracking range shifts in response to climate change. Incorporating these considerations will enhance the utility and efficacy of eDNA metabarcoding for routine biomonitoring applications.

## Introduction

Over the past decade, the use of environmental DNA (eDNA) to survey biological communities has grown tremendously (Taberlet et al., 2018; Beng & Corlett, 2020; Miya, Gotoh & Sado, 2020). These eDNA approaches identify individual species or reconstruct entire communities from DNA shed by resident organisms into the environment (*e.g.*, seawater, freshwater, soil, or air) (Thomsen & Willerslev, 2015; Barnes & Turner, 2016; Deiner et al., 2017; Harrison, Sunday & Rogers, 2019; Tsuji et al., 2019; Clare et al., 2022; Lynggaard et al., 2022). A key strength of eDNA is its versatility and applicability across any terrestrial or aquatic ecosystem and across the tree of life from microbes to mammals (Stat et al., 2017; Djurhuus et al., 2020; Chavez et al., 2021). Yet, despite the value of eDNA for biomonitoring and the rapid adoption of this approach within the academic community, limited experience and expertise in molecular ecology by resource managers inhibits the application of eDNA in resource management.

Harnessing the power of eDNA for ecosystem monitoring and management requires that resource managers understand the value and accessibility of such approaches as well as key methodological choices underpinning eDNA approaches to develop effective molecular biomonitoring strategies. For example, quantitative polymerase chain reaction (qPCR) assays (Yamahara et al., 2019) (see also ddPCR assays (Lafferty et al., 2018) or LAMP (Williams et al., 2017)) selectively identify and quantify individual target species within an eDNA sample, and are particularly useful for detecting presence/absence of rare and invasive species (Biggs et al., 2015; Evans et al., 2017; Beng & Corlett, 2020). Single species eDNA approaches have robust Standard Operating Procedures (SOPs) (Abbott et al., 2021), are routinely used in legal and regulatory frameworks (Mahon et al., 2013; Wilcox et al., 2013; Biggs et al., 2015; Thomas et al., 2020), and are particularly effective at detecting rare and invasive species in aquatic habitats (Larson et al., 2020; Morisette et al., 2021). In contrast, metabarcoding ascertains the species composition of an ecosystem (Beng & Corlett, 2020), differentiating communities inhabiting different microhabitats (Port et al., 2016; Monuki, Barber & Gold, 2021), monitoring local ecosystems (Stat et al., 2019; Postaire et al., 2020) or testing the impact of management strategies (Gold et al., 2021b) while still providing information on invasive, protected, and rare species (Kelly et al., 2014; Bohmann et al., 2014). Although a growing number of review papers (Deiner et al., 2017; Taberlet et al., 2018; Beng & Corlett, 2020) provide an extensive historical summary and overview of considerations for the design and implementation of eDNA metabarcoding, these papers are written for molecular ecologists and are less accessible to resource managers without specialized molecular lab experience.

This article is designed to help managers without molecular ecology experience understand how methodological considerations of eDNA metabarcoding impact results, so that eDNA data can be collected and used appropriately, with the greatest impact for management efforts. We explicitly highlight five key considerations for designing a metabarcoding survey to help resource managers maximize the benefit of eDNA metabarcoding data as a complement to conventional field methods (summarized in Table 1). We then present a case study, applying these considerations to eDNA monitoring of one of the world’s busiest shipping ports.

**Table 1:** Key considerations for metabarcoding studies. These five considerations are recommended as important steps prior to deploying an eDNA metabarcoding approach for resource monitoring.

**Selection of Genes and Primers**
No single gene can capture all species diversity. Primer selection(s) will vary depending on (1) species of interest and (2) sufficient variability in the gene(s) to distinguish target species. Benchmarking primer sets and genes both in the lab and bioinformatically is important to determine an assay’s specificity and breadth.
**Barcode reference database**
Matching reference sequences for each target species and for the barcoding gene(s) of interest are needed to ensure accurate species level identification. These sequences can be obtained from public sequence databases such as GenBank, BOLD, *etc.*, or can be generated from reference tissue voucher specimens of target species.
**Apply Decontamination Pipelines**
Controls for field, lab extraction, and amplification are necessary to identify contaminants. Decontamination pipelines are needed to distinguish signal and noise from raw eDNA sequence calls. Site occupancy modeling of sequence co-occurrence patterns enhances the interpretation of eDNA data.
**Pilot study to define spatial/temporal variance**
eDNA sampling plans must be designed with specific monitoring questions in mind. A pilot study will be needed to define the level of spatial and temporal variance of eDNA in your system.
**Archiving of samples, extracts, and raw sequence data**
Like a time capsule, eDNA samples are an invaluable record of the past that can be reanalyzed as new technologies develop. Even without physical reanalysis, improved barcode libraries and analytical algorithms allow metabarcoding sequence data to yield improved results over time.

We note that our article does not cover the full scope of molecular methodological choices that impact the efficacy of eDNA metabarcoding because (1) we feel that the published literature has discussed such comparisons at length and (2) such detailed discussion of molecular protocol optimization is better suited to molecular scientist practitioners as opposed to marine resource managers, the target audience of our manuscript. Previous work has demonstrated a suite of laboratory and field choices that can impact downstream eDNA metabarcoding results and interpretation including sample collection type (Holman et al., 2019; Koziol et al., 2019), filter type (Turner et al., 2014; Spens et al., 2017), sample preservation method (Mauvisseau et al., 2021), DNA extraction method (Deiner et al., 2018; Pawlowski et al., 2021), PCR methods (Pawluczyk et al., 2015; Kawato et al., 2021), and library preparation methods (Bohmann et al., 2021; Zaiko et al., 2022). We refer readers interested in the optimization of such molecular approaches to the references above.

Here we focus on the five following considerations:

### Selection of genes and primers

Whether SCUBA surveys, benthic trawls, or eDNA metabarcoding, a key step of any biomonitoring program is identifying target taxa, as this choice directly informs the sampling methodologies. For eDNA metabarcoding, the choice of target taxa directly informs the selection of metabarcoding loci, the “molecular net”, used to capture and catalog local diversity (Shelton et al., 2019). The process of DNA sequencing and bioinformatic analysis of these loci produces a table of dereplicated (*i.e.*, grouped similar sequences) DNA sequences. There are two main approaches to dereplicate sequences: (1) amplicon sequence variants (ASVs) which are unique DNA sequences determined using Bayesian probabilities of known sequencing error and (2) operational taxonomic units (OTUs) which are clustered sequences determined using an arbitrary sequence similarity (*e.g.*, 97%, which corresponds to a gross average genetic sequence difference across all life) (Antich et al., 2021). Depending on the underlying variability of the barcoding locus, multiple distinct ASVs can be assigned to the same species (Gold et al., 2021a) or multiple species can be assigned to the same ASV (Min, Barber & Gold, 2021).

Importantly, no one “universal” metabarcoding locus can provide species resolution across the entire tree of life (Taberlet et al., 2018; Curd et al., 2019). Instead, different loci are better suited for some taxa than others. As such, monitoring projects need to decide which target taxa are most important to capture (*e.g.*, all animals or fishes) and to what taxonomic resolution (*e.g.*, genus or species level) to strategically inform locus and primer selection. Alternatively, one can use multiple barcoding loci to gain the desired taxonomic coverage (Arulandhu et al., 2017; Stat et al., 2017; Holman et al., 2021), although laboratory costs increase with the number of loci added. Clearly defining the target taxa allows for choosing the minimum number of target loci to adequately distinguish the intended biomonitoring targets (Kelly et al., 2014).

Benchmarking of each metabarcoding assay is a critical step to determine how well it can identify your target species (Edgar, 2018a; Gold et al., 2021a). Specifically, we refer to benchmarking as the cross validation of a specific primer set and PCR protocol both in the lab and bioinformatically to determine the taxonomic breadth and resolution for target taxa (Edgar, 2018b; Curd et al., 2019). The significant effort, time, and cost that goes into robustly developing and benchmarking a metabarcoding assay means that managers designing biomonitoring studies should carefully consider the pros and cons of choosing loci that are not well documented and validated in the peer reviewed literature (McGee, Robinson & Hajibabaei, 2019; Schenekar et al., 2020). See Bruce et al. (2021) for a publicly available practical guide.

### Barcode reference database

Key to the success of any metabarcoding effort are the employed reference barcode databases, the “Rosetta stone” that connect the unique DNA sequences to the species names of organisms shedding eDNA into their environment (Coissac et al., 2016). High quality databases are based on specimens expertly identified to species based on morphology, have tissues sequenced at relevant barcoding loci, and have the sequences deposited in a publicly available genetic database (*e.g.*, NCBI GenBank or Barcode of Life Database [BOLD]). These barcode reference sequences can then be readily used to identify unknown ASVs to species (Moritz, Cicero & Godfray, 2004; Camacho et al., 2009; Ward, Hanner & Hebert, 2009; Bergsten et al., 2012; Benson et al., 2018; deWaard et al., 2019; Gaytán et al., 2020). When species are missing in the barcode reference database, sequences from those species in eDNA will at best be unresolved to species level or, at worst, may be positively assigned to the wrong species because of “best match” algorithms (Curry et al., 2018; McGee, Robinson & Hajibabaei, 2019; Schenekar et al., 2020; Stoeckle, Mishu & Charlop-Powers, 2020). As such, more complete and comprehensive barcode reference databases increase the resolution of taxonomic identifications (Schenekar et al., 2020; Gold et al., 2021a).

Although generating high quality reference barcode databases is critical, it takes significant time and effort to generate and curate additional species barcodes across the tree of life (∼$20 USD per species per barcode and ∼250 h effort for 756 fishes in an established molecular laboratory in Gold et al. (2021a) and Gold et al. (2021b)) (Leray et al., 2012), not to mention the costs of specimen collection or long-term voucher specimen archiving. Fortunately, regional and global efforts have been underway for the past two decades to generate comprehensive reference databases, and hundreds of thousands of reference sequences are already available through publicly available genetic databases (Benson et al., 2000; Ratnasingham & Hebert, 2007). However, reference databases are far from complete for many taxonomic groups with vast differences in coverage across the tree of life (McGee, Robinson & Hajibabaei, 2019). Thus, primer selection decisions should also consider the completeness of reference barcode availability and the costs associated with generating additional barcodes for a novel primer set of interest within your study system.

### Apply decontamination pipelines

Next generation sequencing approaches produce millions to billions of sequences in a single sequencing run (Singer et al., 2019; McClenaghan, Compson & Hajibabaei, 2020), so sequencing errors, even at low frequency, have the potential to impact the interpretation and understanding of eDNA metabarcoding signatures (Furlan, Davis & Duncan, 2020). Thus, pre-processing sequence data through bioinformatic and decontamination pipelines is essential to remove low quality data and improve interpretation of results (Goldberg et al., 2016; Kelly, Gallego & Jacobs-Palme, 2018; McKnight et al., 2019; Gallego et al., 2020).

Although many decontamination pipelines focus on removing low quality DNA sequences and clear contaminants (*e.g.*, turkey DNA in a kelp forest), an important advancement in eDNA metabarcoding is the application of site occupancy modeling frameworks. These approaches use the co-occurrence of ASVs across technical PCR and field sample replicates to estimate detection probabilities, and thus provide a metric of confidence of detection for a given species at a particular locality (Schmidt et al., 2013; Chambert et al., 2018; Doi et al., 2019; McClenaghan, Compson & Hajibabaei, 2020). Although not typically applied to decontamination pipelines, quantifying occupancy probability for individual species/ASVs can establish defensible thresholds for retaining only high confidence sequence detections (*e.g.*, a sequence is detected in two-thirds of replicates) (Gallego et al., 2020). Given that errors do occur in molecular preparation and sequencing, the application of a site occupancy framework allows for the objective and repeatable discrimination of signal from noise (Gallego et al., 2020). Importantly, this approach removes ASVs with low confidence detections (noise) which may arise from sequencing errors or from rare targets (Schmidt et al., 2013; Chambert, Miller & Nichols, 2015; Chambert et al., 2018). Thus, such occupancy-based decontamination frameworks increase the reliability and interpretation of eDNA metabarcoding results (Goldberg et al., 2016).

### Pilot study to define spatial and temporal variance

Before beginning to implement eDNA assays for routine biomonitoring, it is advisable to conduct a pilot study to establish the temporal or spatial variance of eDNA signatures (Goldberg et al., 2016). Of particular concern in marine environments are transport dynamics (Barnes & Turner, 2016) that may integrate eDNA signals over broader spatial scales in open ocean and nearshore tidally influenced systems (Yamamoto et al., 2017; Kelly, Gallego & Jacobs-Palme, 2018; Andruszkiewicz et al., 2019; Jeunen et al., 2019; Jeunen et al., 2020a; Fremier et al., 2019; West et al., 2020; Andruszkiewicz Allan et al., 2021; Lafferty et al., 2021). The pilot study should be large enough to properly identify target organisms, quantify the spatial or temporal variance in the eDNA distribution, and be as representative as possible of the ecosystem being monitored, while taking into account field and financial constraints (Taberlet et al., 2018). Understanding the spatial and temporal scale in which eDNA signatures persist is vital for determining the sampling intensity needed to adequately measure monitoring targets (Thomsen & Willerslev, 2015; Carraro, Stauffer & Altermatt, 2021). Sampling intensity is further shaped by the goals of the monitoring program (Deiner et al., 2017). For example, monitoring baseline values of key indicator taxa (Ballare et al., 2022), or community turnover (Lin et al., 2021) will require much less sampling effort than programs focused on quantifying total biodiversity and/or rare and elusive taxa (Gold et al., 2021b; Lamy et al., 2021) or sampling exceptionally high diversity ecosystems (Juhel et al., 2020; Madduppa et al., 2021; Marwayana et al., 2022).

### Archiving of samples, extracts, and raw sequence data

eDNA samples represent biodiversity snapshots at a particular location and time (Yamamoto et al., 2016) and, if preserved, can provide insights analogous to the kinds of rich data provided by permanently archived voucher specimens in museums. Both environmental samples and eDNA extractions have value as curated research collections and require storage in appropriate, long-term repositories. These collections can be used in the future to test new or improved assays and sequencing technologies (Jarman, Berry & Bunce, 2018; Singer et al., 2019; Meyer et al., 2021). Permanent physical repositories for eDNA are only slowly becoming available as, at present, neither universities nor natural history museums have developed standardized accessioning protocols for long-term caretaking of eDNA materials (Deck et al., 2017; Jarman, Berry & Bunce, 2018). However, archiving of physical samples and DNA extractions requires financial investment in archival systems and freezer space.

In addition, archived sequence data can be re-analyzed as analysis methods improve and new reference sequences are added to the barcode databases (Gold et al., 2021a). Reanalysis can be performed at any time without any further lab work as long as sufficient metadata are associated with the sequence data. Therefore, all sequence read data should be deposited in the NCBI Sequence Read Archive or similar repositories with metadata documentation to allow for data permanence, reproducibility, and reanalysis (Benson et al., 2018; Leray et al., 2019). If needed, supplemental metadata exceeding what NCBI accommodates should be archived in stable long-term DOIs such as through Dryad (datadryad.org). The eDNA community has yet to settle on minimum metadata reporting requirements, leading to inconsistent reporting (Nicholson et al., 2020; Nicholson et al., 2020), but a number of useful guides and ontologies exist, including MiXs (http://w3id.org/mixs) and DarwinCore (https://dwc.tdwg.org/).

### Case pilot study at the Ports of Los Angeles and Long Beach

**Figure 1:**
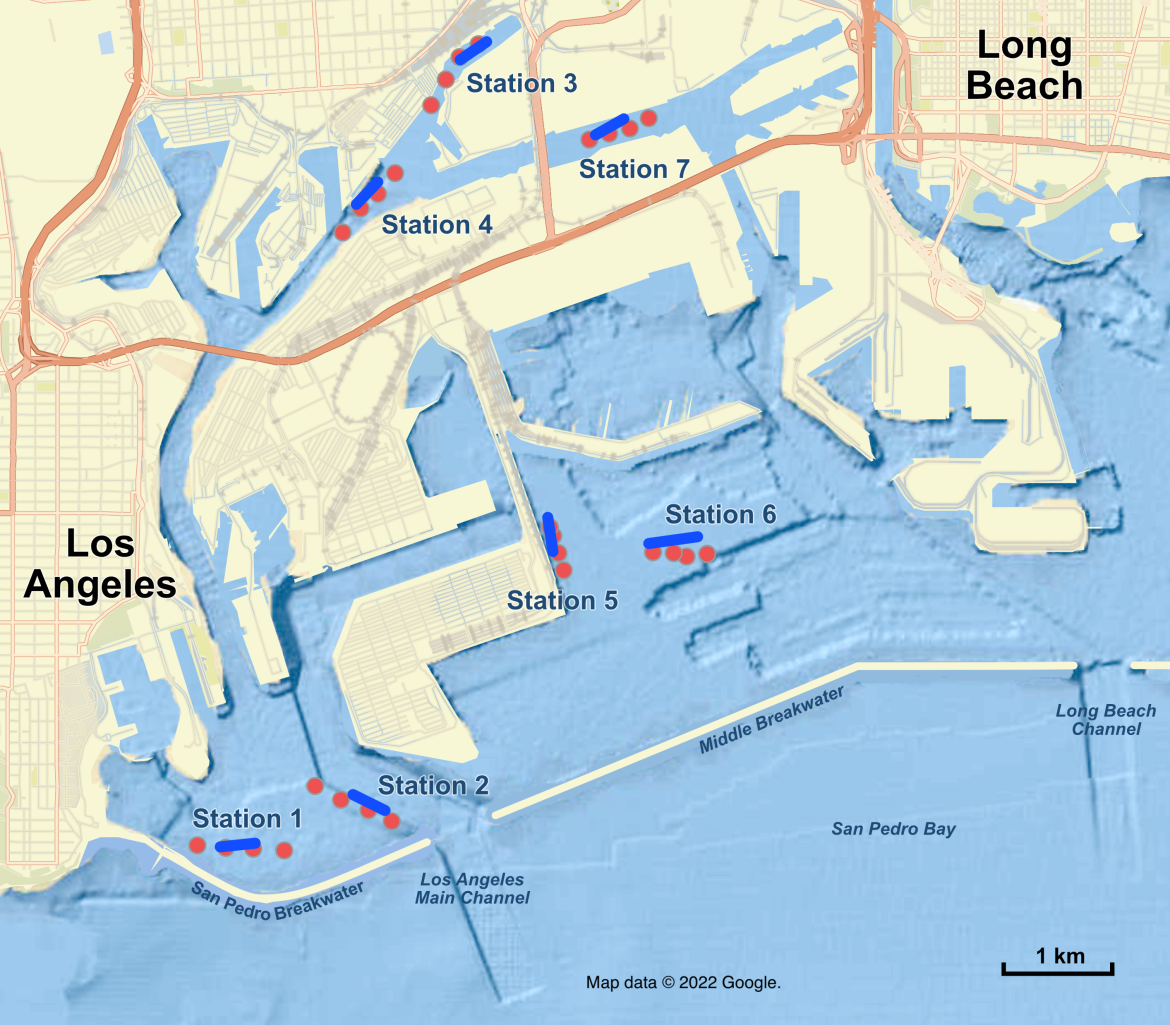
Map of sampling stations in the Port of Los Angeles and Port of Long Beach. Red dots are the four eDNA sampling locations at each station; blue lines are trawl tracks at each station. Map data ©2022 Google.

**Figure 2:**
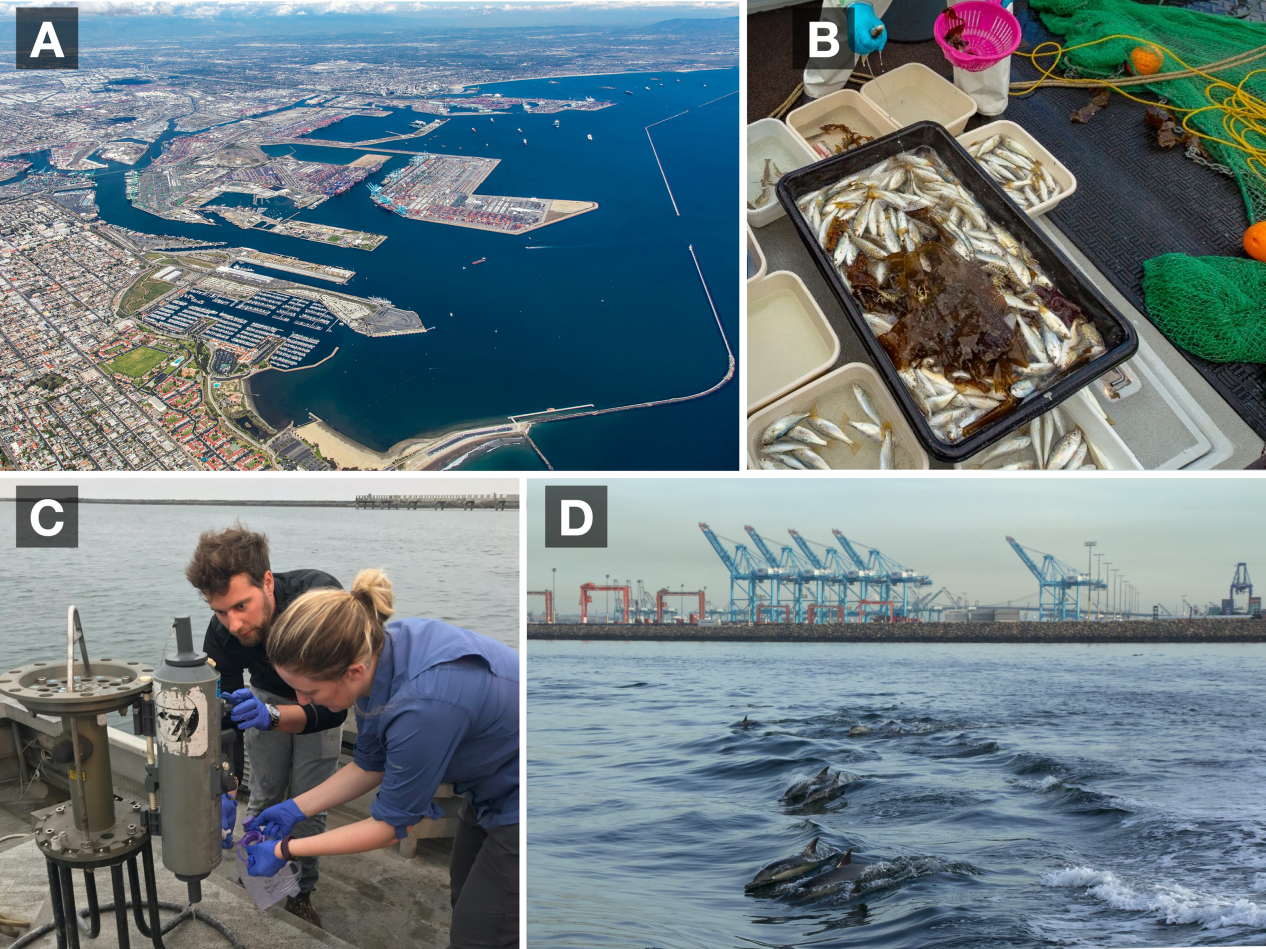
Long Beach-Los Angeles Port Complex biomonitoring surveys. Aerial photograph of the Long Beach-Long Angeles Port Complex, one of the world’s largest commercial ports (A). Results of a midwater trawl for fish biomonitoring within the Port complex (B). Field sampling of seawater for eDNA monitoring. The transferring of seawater from a Niskin into a sterile 1 L pouch is depicted (C). Common dolphins (*Delphinus delphis*) are one of the thousands of marine species that utilize the marine habitats of the Port complex (D). Photo Credits: (A) Port of Los Angeles, (B) Wood Environment and Infrastructure, Inc., (C) Janie Chen, and (D) Zachary Gold.

To highlight the value of eDNA for monitoring, and illustrate the impact of considerations outlined above, here we present a case pilot study from one of the world’s largest commercial port complexes: the combined Ports of Los Angeles and Long Beach, California. The Long Beach-Los Angeles Port Complex (Figs. 1 and 2) is defined by dredged channels and basins with much of the shoreline protected by rock structures, concrete bulkheads, and wharves and piers; almost none of the original marine and coastal habitats prior to the construction of the port remain. The ports are charged by various laws both with accommodating maritime commerce, navigation, and fisheries and with managing and protecting the marine resources of the harbors for the people of California. As part of that stewardship, the ports have conducted periodic, jointly funded comprehensive biological surveys since 2000 to assess environmental impacts of port activities and development, compare current biological communities with historical surveys spanning as far back as 70 years, as well as to monitor for the introduction of non-native marine species (MEC, 2002; Science Applications International Corporation, 2010; MBC, 2016; Wood & IS, 2021). These biological surveys are conducted periodically (*e.g.*, 2000, 2008, 2013, 2018), and involve significant effort to characterize soft bottom, hard substrate, and pelagic habitats of the entire Port Complex. The 2018 survey included ichthyoplankton net tows, visual scuba surveys, invertebrate quadrat surveys, benthic otter trawls, lampara net, beach seines, and visual marine mammal and bird surveys (Wood & IS, 2021), and required 7,500+ person-hours at a total program cost of $1.3 million USD. Across all sampling methods, the 2018 Biosurvey identified 104 species of fish (87 adult species and 17 larval taxa), 859 invertebrate taxa, 40 algae taxa, 87 species of birds, and five species of marine mammals totaling over 1,000 taxa living throughout the San Pedro Bay harbors, the highest diversity recorded in the four complex-wide Biosurveys conducted.

In this study, we compare and integrate the results of only the contemporaneously conducted conventional benthic otter trawls for fish with paired eDNA metabarcoding to evaluate the extent of complementarity between survey methods and let us hone future eDNA metabarcoding sampling designs within this port system and coastal systems across the Southern California Bight and California Current Large Marine Ecosystem. These comparisons highlight the value of eDNA for monitoring and the tradeoffs associated with different approaches to the five methodological considerations outlined above.

## Materials & Methods

**Figure 3:**
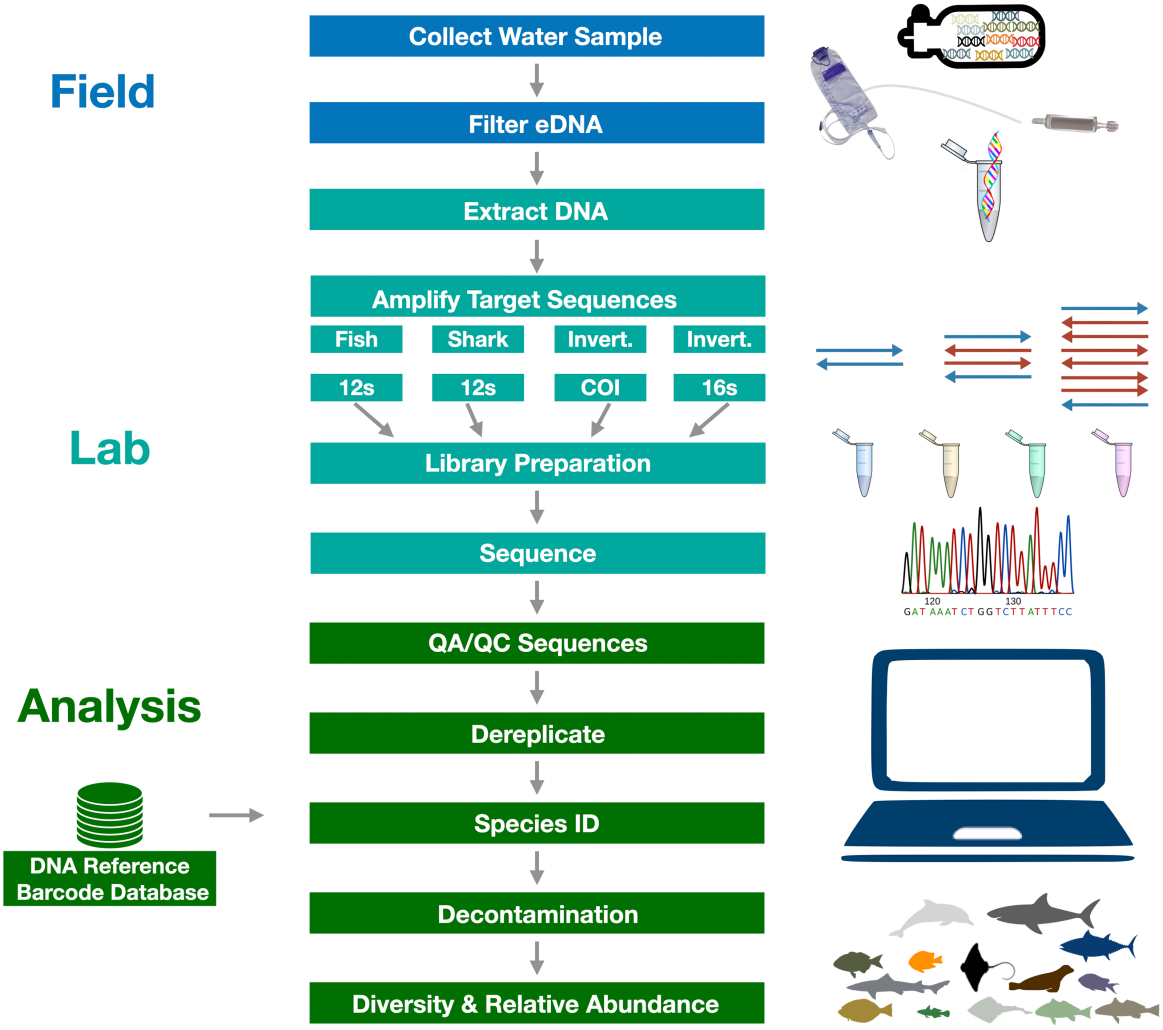
Graphical summary of eDNA metabarcoding methods used in the Ports Complex pilot study. A full detailed description of the methods is provided in Appendix 1.

The sequence of procedures followed in the eDNA sample collection, sequencing, and analysis is summarized in Fig. 3. We present a summarized description of the methods here as this is not the focus of our manuscript, but provide a full detailed description of the methods in Supplemental Materials Appendix 1. All sequences are made available through GenBank SRA (PRJNA851175), data through Dryad (https://doi.org/10.5068/D1B10H), and code through Zenodo (https://doi.org/10.5281/zenodo.6667975). All work was conducted under State of California permits (SC-004668 and S-191440006-19209-001).

### Field sampling: environmental DNA

To compare the efficacy of eDNA and trawling for monitoring of fish communities in the Ports of Los Angeles and Long Beach, we conducted paired eDNA and trawl surveys at seven stations on 20–21 August 2018, each of which is a standard trawl station in the conventional periodic biodiversity surveys conducted by the Ports (Table 2, Tables S1, S2, Figs. 1 & 2C; full methods are detailed in Supplemental Materials Appendix 1). We sampled three replicate 1 L seawater samples at four locations 2 m above the sea floor at each of the 7 sampling stations along an approximately 150 m transect aligned with the trawl track, using the R/V Yellowfin as a sampling platform. Seawater was gravity filtered onto 0.22 µm Sterivex filter cartridges and stored on dry ice before being transferred to a −20 °C freezer.

### Field sampling: Midwater Trawls

Within 15–90 min of the eDNA seawater sampling, we trawled the station for 5 min with a 7.6 m semi-balloon otter trawl with 2.5-cm side mesh and 1.3-cm mesh cod-end deployed from the R/V Early Bird II (Fig. 2D), following standard protocols of the Ports Biosurvey program, as detailed in the 2018 Biosurvey report (Wood & IS, 2021). All fish were identified to species level and standard length was recorded. See Table S2 for details of trawl locations and depths.

### Environmental DNA extractions, amplification, and sequencing

We exacted eDNA from the frozen Sterivex filters within one week of collection using the DNeasy Blood and Tissue Qiagen Kit (Spens et al., 2017). We amplified eDNA with four primer sets (described below) modified with Illumina Nextera XT adapters (Illumina, San Diego, CA) in triplicate PCR replicates. We prepared sequencing libraries following the methods of (Curd et al., 2019) which involved a second PCR indexing step to tag libraries so they can later be distinguished from each other (See S1 Supplemental Materials Appendix 1). Resulting indexed libraries were bead cleaned to remove fragments <200 bp, were quantified with the Qubit BR assay (Thermofisher, Waltham, MA, USA), then sequenced on an Illumina MiSeq (Illumina Inc., La Jolla, CA, USA) with V3 kit to obtain paired-end 2 × 300 bp reads at the Technology Center for Genomics & Bioinformatics (UCLA, CA, USA) (See S1 Supplemental Materials Appendix 1 for further details).

**Table 2:** Case study eDNA sampling design.

**Sampling Level**	**Sample Enumeration**	**Total Number**
Stations	1, 2, 3, 4, 5, 6, 7	7 stations
Locations (4 per station)	(4 locations ×7 stations)	28 locations
Samples (3 per location)	(3 samples ×28 locations) + (1 blank ×7 stations)	84 samples + 7 blanks

### Bioinformatic analysis and taxonomic assignment

We used the *Anacapa Toolkit* (Curd et al., 2019) for amplicon sequence variant (ASV) parsing, quality control, and taxonomic assignment (Curd et al., 2019; Gold et al., 2021a) (See S1 Supplemental Materials Appendix 1 for full description). Importantly, we note that we employed stricter sequence alignment parameters within the *Anacapa classifier* for the *CO1* and *16S* locus (95% identity and query coverage) than the *12S* loci (80% identity and query coverage) given the lack of complete reference databases for the *CO1* and *16S* loci (Curd et al., 2019). Separate reference databases were generated for the *12S, COI,* and *16S* from all publicly available sequencing data in GenBank from October 2019 using *CRUX* with default parameters (Curd et al., 2019). A more comprehensive *12S* reference database was created by supplementing the above database with barcodes from 252 native fish species as detailed in (Gold et al., 2021a).

### Impact of reference databases

To highlight the importance of complete reference databases, we compared MiFish Teleost bony fish sequences by conducting species identification using both the less complete *12S* reference database and the more comprehensive *12S* reference database described above (Gold et al., 2021a; Gold et al., 2021b). For all other comparisons and analyses using the MiFish Teleost bony fish data, we used the more comprehensive *12S* fish reference database. For this study, we did not add additional local or regional DNA barcodes to *16S* and *CO1* reference databases and therefore warn that they are comparatively sparse for native invertebrate species (See Discussion). We chose the above primer sets given their previous application and validation in coastal waters in the California Current and our familiarity using them, recognizing that alternative primer sets could have been used (*e.g.*, *Teleo 12S* or *Evans 16S* primer set; (Kelly et al., 2016; Evans et al., 2016; Andruszkiewicz et al., 2017; Closek et al., 2019; Curd et al., 2019; Djurhuus et al., 2020; Pitz et al., 2020; Gold et al., 2021a; Polanco et al., 2021b).

### Impact of primer selection

To identify the value of including additional markers in eDNA metabarcoding analyses, we compared taxonomic assignments made by each primer set and the incremental value of adding additional barcoding targets. Specifically, we used two fish primer sets: the MiFish Universal Teleost *12S* primer set which is designed to target bony fishes, and the MiFish Universal Elasmobranch *12S* primer set (Miya et al., 2015) which is designed to target sharks and rays. We also included two universal primer sets: the Leray metazoan *CO1* primer set (313 bp) (Meyer et al., 2021), and the Kelly metazoan *16S* primer set (114–160 bp) (Kelly et al., 2016) both designed to broadly amplify all marine metazoans, particularly invertebrates.

### Decontamination pipeline

To explore the importance of decontamination pipelines designed to reduce false positives, we examined the combined fish eDNA data (combined results from both *12S* MiFish primer sets) with and without site occupancy modeling. Decontamination of data including site occupancy modeling was conducted following the methods of Kelly, Gallego & Jacobs-Palme (2018) (See S1 Supplemental Materials Appendix 1).

### Archiving of samples, extracts, and raw sequence data

DNA extracts were archived in the CALeDNA freezer collection at UCLA (Meyer et al., 2021). Raw sequence data was archived on NCBI SRA (SUB11632426). Original filter capsule and water samples were not archived as they were consumed in the DNA extractions.

### Comparison of eDNA metabarcoding and trawl surveys

We compared the fish species identified at each site by conventional trawl survey methods with the combined fish eDNA data, visualizing species detections across methods through Venn diagrams and heat maps.

### Spatial structuring of eDNA

We investigated the variability of eDNA signatures across all locations and stations (Oksanen et al., 2020). We then estimated the sampling completeness, the fraction of diversity observed with our sampling effort, within a given location, within a given station, and within the port as a whole (Hsieh, Ma & Chao, 2016). We did not examine the spatial variation of trawls given the limited sample size (*n* = 7) and lack of within-station replication.

## Results

Across the seven trawl stations in the Ports of Los Angeles and Long Beach conducted between August 20–21, 2018, we captured 452 total individual fish, identifying 17 species belonging to 16 genera and 10 families (Tables S3 & S4).

After processing via the *Anacapa Toolkit* and decontamination, we retained 705 ASVs across 83 samples and 7.1 million reads for the MiFish Teleost primer set, 569 ASVs across 83 samples and 3.2 million reads for the MiFish Elasmobranch primer set, 2,791 ASVs across 79 samples and 3.2 million reads for the Leray *CO1* primer set, and 888 ASVs across 77 samples and 6.9 million reads for the Kelly *16S* primer set (See S1 Supplemental Materials Appendix II).

### Impact of reference databases

Taxonomic assignments of the MiFish *12S* Teleost bony fish primer set using the limited reference database missed 45 native California fish species that were identified using the more comprehensive reference database (Table 3). These species include nine of the 17 fish species observed by the trawl methods (Table S5–S8). We note the four unique CA native fish species identified using the limited database were re-assigned to more ecologically appropriate sister species with higher assignment confidence using the comprehensive database. Given this improved performance of taxonomic assignments, all subsequent analyses used the comprehensive reference database.

**Table 3:** Comparison of reference database completeness. Table of taxonomic assignment metrics made by the limited and comprehensive database on collected eDNA samples. ASVs assigned to each taxonomic rank could not be assigned to lower levels (*e.g.*, assigned to genus could not be assigned to species). Additional Genus and Family level Ids are genera and families that were uniquely assigned by one reference database but not the other. The four unique California native fish species identified using the limited database were re-assigned to more ecologically appropriate sister species with higher assignment confidence using the comprehensive database (Table S5–S8).

**Metric**	**Limited database**	**Comprehensive database**
ASVs Assigned to Species	489	642
ASVs Assigned to Genus	98	38
ASVs Assigned to Family	19	25
Unique Species Identified	65	78
Additional Genus Level Ids	12	6
Additional Family Level Ids	4	3
CA Native Fish Species	34	75
Unique CA Native Fish Species	4	45

### Impact of primer selection

The vast majority of taxonomic assignments were shared between the two *12S* fish primer sets (a total of 76 species representing 70 genera and 45 families). Metabarcoding with the MiFish Elasmobranch primer set produced an additional five species representing five genera and three families, and including White shark (*Carcharodon carcharias)*, Swell shark (*Cephaloscyllium ventriosum*), and Mako shark (*Isurus oxyrhinchus*). The MiFish Teleost primer set uniquely identified two species and no additional genera or families (Tables S5 & S6).

In contrast, we found substantial differences in species observed between the combined fish *12S* primer sets and the *CO1* and *16S* metazoan primer sets. Only three fish species were shared between the *CO1* and the *12S* fish primer sets and only one family level assignment was shared between the universal *16S* and *12S* fish primer sets. Likewise, between *CO1* and *16S* primer sets only 10 species, 12 genera, and 10 families were shared. The *CO1* primer set uniquely revealed a total of 227 species, 204 genera, 165 families, 31 classes, and six phyla. These include a large number of non-animal targets like diatoms, dinoflagellates, and bacteria as well as animal targets such as crinoids, hydrozoans, and polychaete worms (Tables S9 & S10). The *16S* primer set uniquely detected a total of 79 species, 92 genera, and 84 families, eight classes, and three phyla. These were all animal targets including cephalopods, sea stars, sea urchins, cubozoans, and acorn worms (Table S10).

Only 56% of *CO1* ASVs and 32.1% of *16S* ASVs were assigned to species-level resolution, while 92% of both *12S* ASVs were assigned to species-level resolution (Tables S5, S6, S9, & S10). Despite the current reference barcode limitations, *CO1* and *16S* primer sets were able to identify 12 known invasive species actively monitored by the California Department of Fish and Wildlife’s California Non-native Estuarine and Marine Organisms (CAL-NEMO) program (Table S11).

### Impact of decontamination

Use of site occupancy modeling on the combined fish data set removed 42 species that were only observed in a single bottle of water within a given location and at most across 6 bottles of water across the entire study (mean = 2) (Table S12–17). All of these species were native to the region, and they included common fish species like Garibaldi (*Hypsypops rubicundus*) and Rubberlip seaperch (*Rhcochilus toxotes*) as well as unexpected marine mammals including Cuvier’s beaked whale (*Ziphius cavirostris*) and Humpback whale (*Megaptera novaeangliae*) (Table 4).

### Comparison of eDNA metabarcoding and trawl surveys

**Figure 4:**
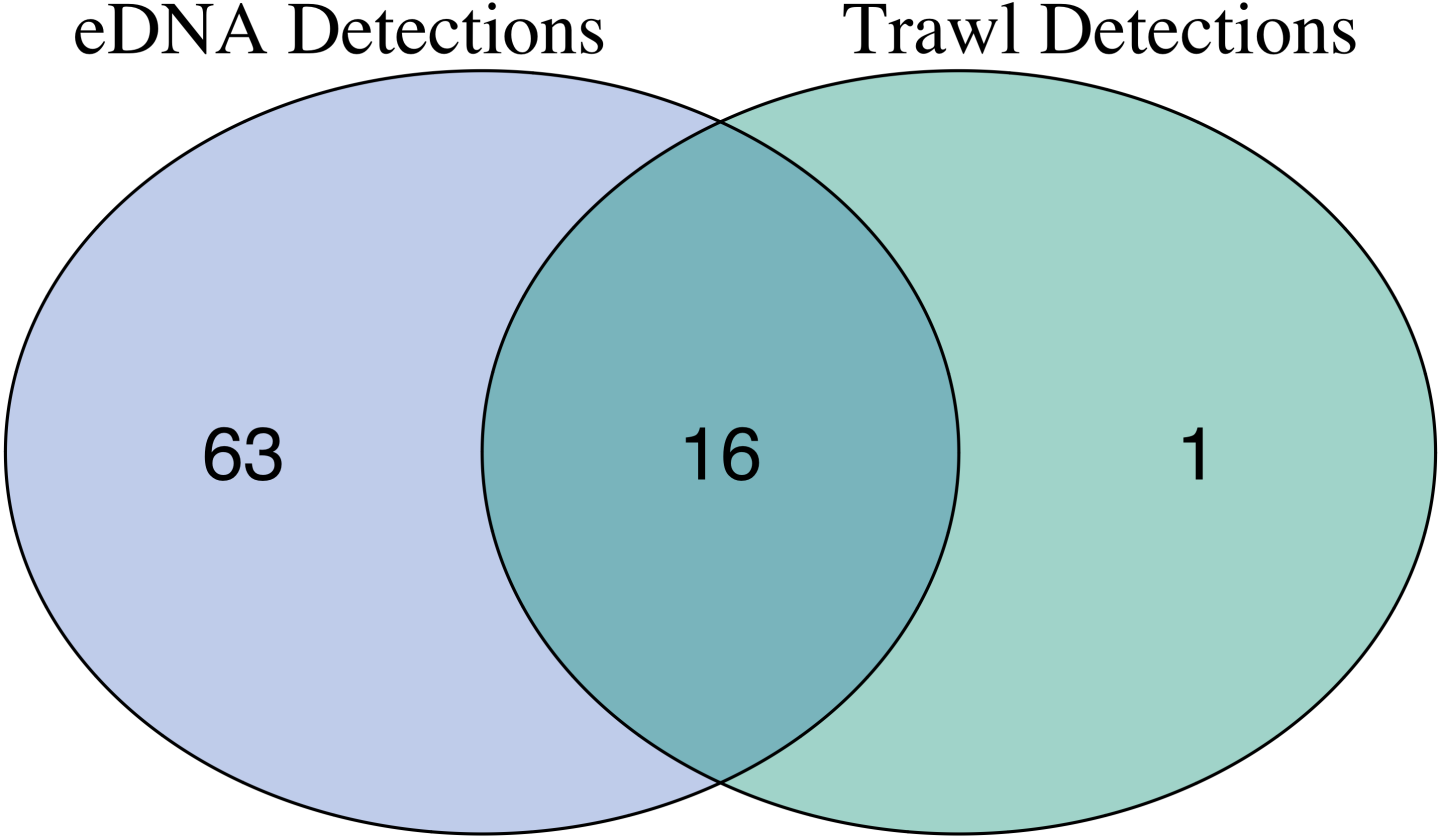
Overlap of fish species detections between trawl and eDNA surveys. Only fish species that passed site occupancy modeling were reported for eDNA surveys. All fish species detected in trawl surveys are reported.

**Figure 5:**
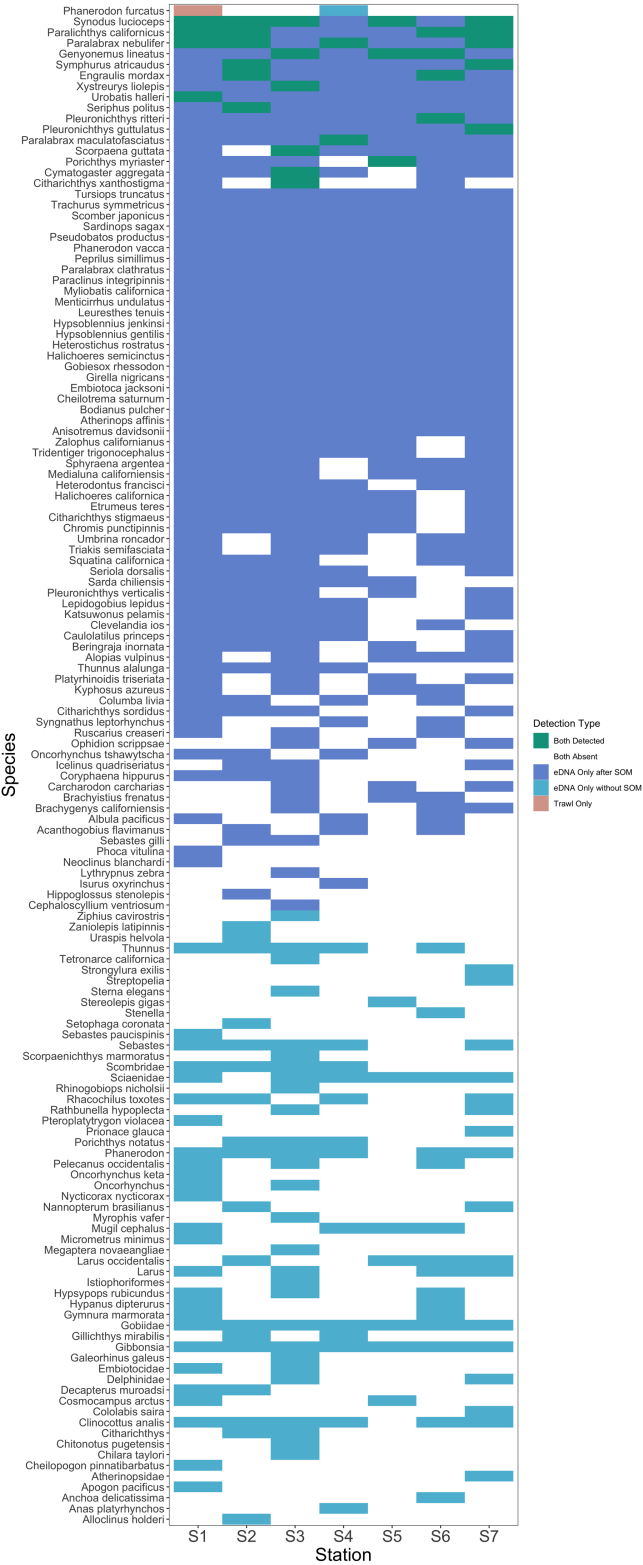
Station-by-station species detections by trawl and eDNA sampling. Tile color indicates whether a species was absent or detected by trawl or eDNA sampling. eDNA only failed to detect a single species at a given trawl station, but did detect that species within the Port complex prior to site occupancy modeling (SOM). In contrast, eDNA more frequently detected all other species observed in trawls and captured a broad range of fish diversity. A substantial number of taxa including many non-native species were detected prior to decontamination with site occupancy modeling.

After site occupancy modeling, eDNA recovered 94.1% (16 of the 17) of fish and elasmobranch species observed across all sampling stations via trawling (Fig. 4; Table S3 & S16). eDNA only failed to detect White seaperch (*Phanerodon furcatus*) for which a single specimen was recovered in one trawl (Fig. 5). However, eDNA did detect this species in a single bottle at a different station prior to the site occupancy decontamination, resulting in low confidence in our detection.

**Table 4:** Comparison of site occupancy decontamination.

**Metric**	**Without site occupancy**	**With site occupancy**
Total Species Identified	125	83
Total Genera Identified	116	77
Total Families Identified	68	49

In addition to the above, eDNA metabarcoding identified 63 species of marine fishes that were not detected in the trawls (Figs. 4 and 5; Tables S3 and S16). Of these species, 55 were fishes native to the California Current Large Marine Ecosystem and two were resident non-native species being monitored by the Ports (Yellowfin goby, *Acanthogobius flavimanus,* and Chameleon goby, *Tridentiger trigonocephalus*). In addition, we detected five commercial fisheries targets that are not frequently observed in California nearshore coastal waters (Table S16). In the absence of site occupancy modeling, eDNA detected an additional 33 fishes native to the California Current.

We note that eDNA also detected three native marine mammals and one native bird not expected to be observed in trawl sampling. In the absence of site occupancy modeling, eDNA detected an additional seven native birds and two native marine mammals (Humpback whale *Megaptera novaeangliae* and Cuvier’s beaked whale *Ziphius cavirostris*).

### Spatial structuring of eDNA

**Figure 6:**
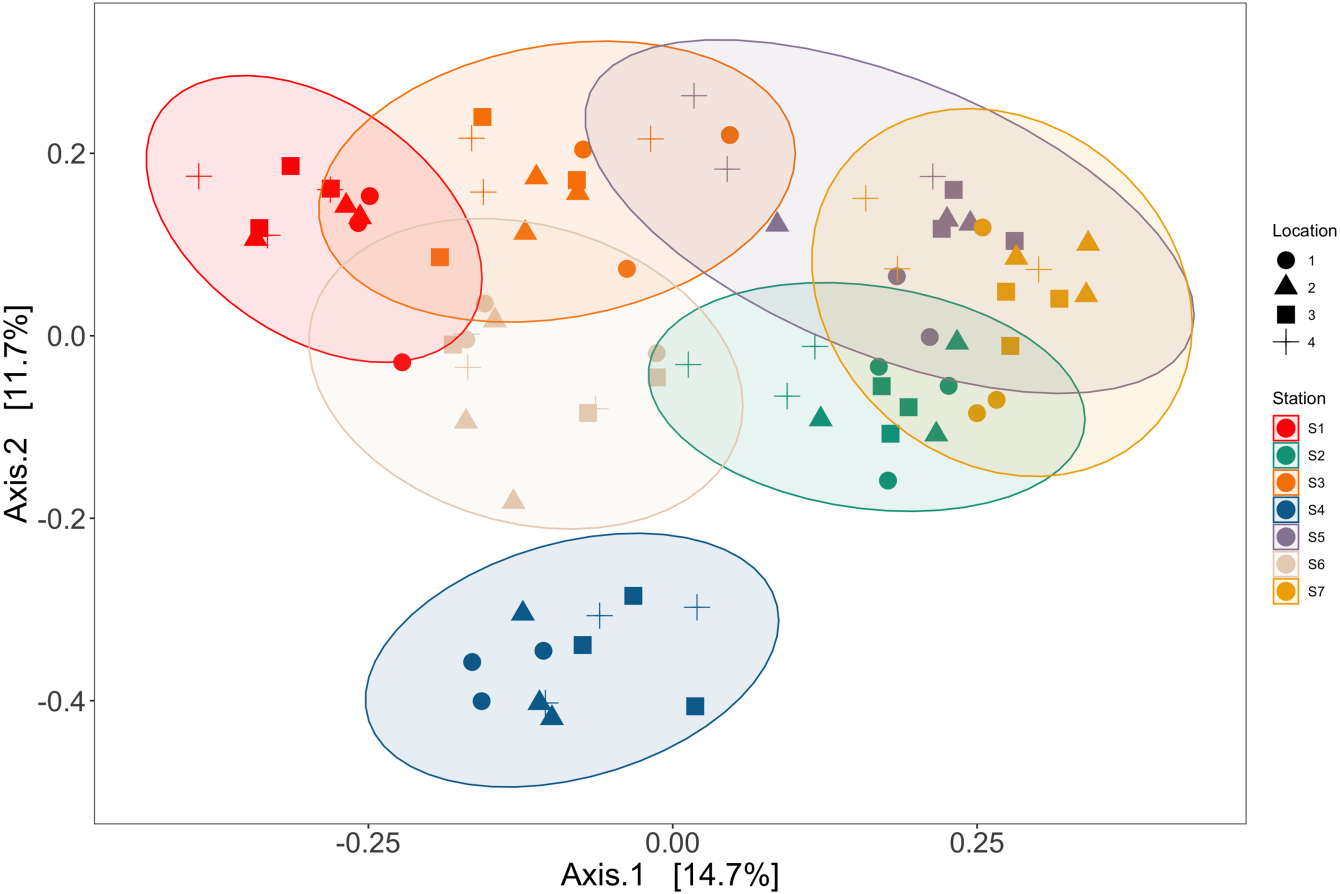
Ordination showing spatial structure between stations and locations. Ordination of principal coordinates analysis revealed clustering within trawl stations (colors) and within locations (shapes) in the Port complex. Station and location explained 36.7% and 20.2% of the variation in vertebrate communities across the Port complex, respectively (PERMANOVA, *p* = 0.001, Table S6).

Station and bottle replicates explained 36.7% and 20.2% of the variation in vertebrate communities across the Port complex, respectively (PERMANOVA, *p* = 0.001, Table S18, See SI Supplemental Materials Appendix II). PCoA ordination demonstrates substantial clustering within stations and bottle replicates within locations in the Port complex (Fig. 6) suggesting bottles sampled at different stations are more different from each other than replicate bottles from the same location.

For both eDNA and trawl surveys, the sample coverage estimate was 99% across all stations within the Port complex, suggesting that seven stations across the Port complex were sufficient to nearly saturate diversity (Table S19). Looking at within-station sampling completeness for eDNA methods, the average sample coverage estimate across locations within each station was 97.5% (90.5–100%, min–max) while across replicate bottles within each location was 94.0% (82.0%–100%). These results suggest that an average of five (4–19) locations within a station, and an average of 7.3 (2–19) bottle replicates within a location, were needed to saturate diversity (Table S20 & S21, Fig. S1–5). However, our results suggest that lower sampling effort–only 20 bottles total out of the 83 taken–are needed to observe the 16 species observed by both eDNA and trawl surveys. In contrast, trawl surveys saturated diversity at six out of seven trawls, suggesting that additional trawl surveys would provide diminishing returns in species recovery and even 50 trawls would fail to detect a substantial portion of the 63 additional fish species detected by eDNA.

## Discussion

Paired comparisons of eDNA and benthic trawls in the Ports of Los Angeles and Long Beach showed that eDNA captured all fish taxa captured in trawls, and all but one species after site occupancy modeling. However, as with previous DNA studies (Thomsen et al., 2012; Kelly et al., 2017; Stat et al., 2019; Jeunen et al., 2020b), eDNA captured substantially more species, capturing more than 4 times as many species as traditional fish trawls. This greater richness was observed even after the application of site occupancy modeling, providing greater confidence in the results (Royle & Link, 2006). Further, eDNA sampling required substantially less risk of habitat disturbance and incidental loss of fish than trawling, making it more amenable to regular sampling, rather than every five years as occurs with current Port Complex and regional monitoring protocols, increasing the ability to understand changes in the Port Complex’s marine ecosystems. Below we summarize the impact of different choices employed in this eDNA pilot.

### Impact of reference databases

Using a more comprehensive database that included DNA barcodes from 252 California marine fishes allows the recovery of an additional 9 species that were also observed in the Port trawl samples and nearly doubles the number of native fish species inventoried (Table 3). The results of the *12S* locus, where high database completeness allowed nearly all ASVs to be assigned to species, stood in sharp contrast to the *COI* and *16S* results, where identification of ASVs to species-level was low.

It is unsurprising that complete reference databases provide the most comprehensive results of community diversity. The major barriers to developing such databases are expense and time (Stoeckle & Hebert, 2008; Ausubel, Trew Crist & Waggoner, 2010; Leray et al., 2012). However, even the less complete databases provided identification of important invasive species. Moreover, to meet the specific needs of resource managers, databases can be augmented to include specific taxa of interest, at relatively minimal expense (*e.g.*, $20/species with access to a standard molecular lab). Furthermore, ASVs can be used to derive taxonomy-free or taxonomy-limited molecular based ecological indices for characterizing the health of marine ecosystems (Aylagas, Borja & Rodríguez-Ezpeleta, 2014; Aylagas et al., 2018; Keck et al., 2018; Pawlowski et al., 2018). As such, while the best results will derive from the most complete databases, incomplete databases should not be viewed as a barrier to incorporating eDNA into monitoring programs.

### Impacts of primer selection

Our success of eDNA metabarcoding for fish inventory results from the selection of metabarcoding loci and primers that had been rigorously benchmarked bioinformatically and in the lab using cross validation by identity approaches as well as tested in the field (Gold et al., 2021a). These results highlight the importance of using validated metabarcoding loci for biodiversity surveys.

Doubling the number of fish primers to target both bony fishes and elasmobranchs (Miya et al., 2015; Miya, Gotoh & Sado, 2020) had marginal benefits in terms of total taxa, expanding taxonomic coverage of our barcoding efforts by only seven species. This result is partially due to the substantial overlap in taxonomic coverage of these primer sets and reference databases despite being designed to preferentially target teleosts or elasmobranchs. Despite a minor increase in total taxonomic coverage, these taxa included species of great potential conservation interest such as White and Mako sharks. Thus, depending on the focus of a specific eDNA metabarcoding monitoring effort, just one primer set may be needed, but the addition of a second primer set (and associated increase of costs) may be justified (Lafferty et al., 2018).

In contrast to the two *12S* primer sets, there was little taxonomic overlap between taxa identified by the *COI* and *16S* primers, both designed to capture broad animal diversity. Moreover, there was nearly zero overlap between the list from *COI* and *16S* results, and the *12S* results. For the *CO1* and *16S* ASVs the relative contribution of library completeness or lack of interspecific variation driving the poor species level assignment is unknown, although no systematic barcoding efforts like Gold et al. (2021a); Gold et al. (2021b) have been conducted for CA coastal marine invertebrates and previous work has moderate species level resolution accuracy of this primer set (Leray et al., 2013; Curd et al., 2019). Despite these limitations, including *COI* and *16S* data allowed for the detection of hundreds of additional non-vertebrate species targets, including 8 invasive species. Thus, if detecting invasive species is a key monitoring goal, using primer sets that cast a wider taxonomical net may be more advantageous. These results highlight the importance of careful selection of primer sets to best support the intended goals of the monitoring efforts.

### Impact of decontamination

Prior to site occupancy modeling, eDNA detected species such as Cuvier’s beaked whale and Humpback whale whose physical presence within the enclosed Port complex is unlikely, and whose detection would be cause for concern as federally protected marine mammals. Site occupancy modeling is critical for distinguishing biological signal from noise by determining whether a detected eDNA signature has a high probability of being a true positive detection or an artifact (Darling, Jerde & Sepulveda, 2021). Because these whales (and other taxa) were not repeatedly detected across multiple bottle replicates at any given station, their true presence in the Port complex has a very low probability. As such, these detections were excluded from subsequent alpha and beta diversity analyses since we could not disentangle such noise from true biological signal.

Such potentially spurious species detections can arise from two predominant causes: sequencing errors or detection of a very low concentration eDNA signal (Furlan, Davis & Duncan, 2020; Silverman et al., 2020). In our study, many of the eliminated detections were consistent with sequencing errors where a small number of sequences from ecologically improbable taxa co-occurred with genetically similar taxa having higher read counts (*i.e.*, *Aotus* monkeys: six reads; humans: 70,676 reads at Station 4). Because these sequencing errors are not consistently found across replicates, site occupancy frameworks can distinguish them from high confidence detections.

In the case of the whales, their detection is more consistent with detecting a low concentration eDNA signal. eDNA concentrations are a function of the number, biomass, and DNA shedding rate of a species (Barnes & Turner, 2016; Harrison, Sunday & Rogers, 2019), making rare, small, and low-shedding species more difficult to detect with eDNA metabarcoding (Bessey et al., 2020; Di Muri et al., 2020; Egozcue et al., 2020; Silverman et al., 2020). Additionally, laboratory and modeling studies of eDNA generation, degradation, and transport suggest that eDNA can travel tens of kilometers in the marine environment (Andruszkiewicz et al., 2019), but becomes diluted with time and distance. Thus all else being equal, species closer in proximity to the sampling location are believed to contribute a higher proportion of total DNA to an observed eDNA signature (Ely et al., 2021; Monuki, Barber & Gold, 2021). Given that these whale sequences were only detected at Station 1, near the opening of the Main Channel to the open ocean, the most likely explanation for their detection is eDNA transported into the Port from offshore where both species are regularly observed.

In another example, our trawl surveys only detected a single individual of White seaperch (*Phanerodon furcatus*) across all stations, suggesting this species is rare and at relatively low biomass within the Port Complex. Given this, we would hypothesize that the concentration of White seaperch eDNA is quite low within the Port Complex and that we would expect fewer bottle replicates to detect this species as observed here (replicate detections = 1, read counts = 29). In contrast, all other 16 concurrently detected species were observed in multiple bottle replicates and locations within each station. These results are consistent with the hypothesis that greater abundance and biomass of target species leads to higher concentrations of eDNA and thus have a greater probability of detection across bottle replicates.

Whether site occupancy modeling is employed in an eDNA monitoring program will depend on the goals. If the goal is to document the maximum total biodiversity, and the integration of eDNA signals from nearby habitats is acceptable, the site occupancy modeling may not be necessary, although excluding non-native species like *Aotus* monkeys through other decontamination approaches will still be required. However, if the goal is to have high confidence detections from a specific area, then site occupancy modeling is essential. Importantly, site occupancy decontamination can be calibrated and adjusted to the monitoring objectives using a critical ecological lens (Chambert et al., 2018; Darling, Jerde & Sepulveda, 2021). In some cases, it may even be useful to examine the data with and without site occupancy modeling to differentiate frequently occurring taxa within an ecosystem from more transient species (Monuki, Barber & Gold, 2021).

### Impact of sampling design

Results showed significant differences in eDNA signatures across sampling stations, highlighting the ability of eDNA metabarcoding to distinguish marine communities in the Ports of Los Angeles and Long Beach on the scale of ∼50 m, similar to previous nearshore coastal studies (Port et al., 2016; Yamamoto et al., 2017; O’Donnell et al., 2017; Kelly, Gallego & Jacobs-Palme, 2018; Jeunen et al., 2020a; Ely et al., 2021; West et al., 2021). Importantly, eDNA differentiated among these habitats with as few as a dozen spatially structured samples per site (Fig. 5).

Sampling was sufficient to differentiate among sampling stations, but would have needed at least 15 additional locations per station and an additional 15 bottle replicates per location to completely saturate species diversity. Yet, the sampling design achieved >95% sample coverage with four locations per station and ∼90% sample coverage with three bottle replicates, capturing the vast majority of fish diversity within the Port complex. Furthermore, given that we observed the greatest dissimilarity between bottles collected at different sampling locations, our results strongly suggest that future sampling efforts targeting a greater number of stations with fewer replicates per station would better maximize fish diversity with the same number of samples.

Unfortunately, it is not possible to generalize sampling protocols based on our results. Factors such as water velocity, temperature, UV, microbial activity, and pH all influence eDNA degradation rates (Barnes et al., 2014; Strickler, Fremier & Goldberg, 2015; Shogren et al., 2017; Harrison, Sunday & Rogers, 2019). Thus, the spatial scale of eDNA metabarcoding signatures is determined by the fate and transport of eDNA in the local environment, and these factors differ dramatically across habitats and ecosystems (Barnes & Turner, 2016). For example, if we were to repeat this pilot study in a coastal marine environment with large tidal exchange or swift ocean currents, we would have spread our sampling efforts over a greater spatial area (*e.g.*, fewer locations sampled per station and more stations sampled total) given the increased capacity for transport and mixing. Ultimately, pilot studies are essential to quantify the spatial and temporal structure of eDNA signatures in the environment being monitored, relevant to the biomonitoring questions of interest.

### Impact of archiving

Although the *CO1* and *16S* data lacked complete reference databases, resulting in poor taxonomic resolution for the majority of our environmental sequences, an important advantage of eDNA is that the data can be reanalyzed as reference databases improve. Thus to enhance the existing barcode database for local invertebrates, we initiated the “Los Angeles Urban Ocean Expedition” in 2019. This two-week marine invertebrate “bioblitz” involved more than twenty taxonomists, varied sampling technologies, and identified over 3,000 specimens representing more than 1,200 taxa (https://research.nhm.org/disco/lauoe2019/). Reference barcodes from these specimens are presently being generated and posted to BOLD (Ratnasingham & Hebert, 2007) and GenBank (Benson et al., 2018). We will reevaluate the eDNA analysis of the invertebrate and algal community once the barcode database is populated with the above barcoding efforts, allowing for benchmarking and validation for key invertebrate taxa of interest.

Much like traditional museum collections, eDNA samples can be archived as a record of what was present at a particular location at a particular moment in time. However, a key advantage is that these samples are a permanent snapshot of entire marine communities from microbes to metazoans. As novel primer sets are developed, and as new questions arise, archived eDNA samples can be queried, allowing researchers to re-enter a past ecosystem to ask questions like, when a particular invasive species was first detected, when a rare or endangered species was last detected, or whether a pathogen has always been present in the ecosystem, etc.

### Advantages and shortcomings of eDNA metabarcoding

eDNA metabarcoding is one of many survey methods that can support marine ecosystem management. Deciding which method(s) to deploy depends on the biomonitoring objectives, methodological costs and biases, and the strengths of the chosen methods, while accounting for their weaknesses.

The strengths of eDNA include its high sensitivity, wide taxonomic breadth, takeless nature, relative cost effectiveness, and ability to archive samples for reanalysis. Consistent with previous studies (Thomsen et al., 2012; Port et al., 2016; Goodwin et al., 2017; Stoeckle et al., 2021), eDNA recovered nearly all the fish diversity reported by near-simultaneous trawls while also detecting four times the number of fish species identified in the trawls. Moreover, by adding additional primer sets, we identified species of special conservation interest, as well as invasive fish, invertebrate, and algae species under surveillance by the California Department of Fish and Wildlife in the Port complex–and with just a few liters of water, without harming a single animal. In low visibility environments like the Ports of Los Angeles and Long Beach, targeted eDNA protocols focused on detecting a single (or few) invasive species will likely be more effective than putting SCUBA divers in the water. Because eDNA sampling generally requires fewer people and field time it can be more cost effective compared to traditional surveys, allowing for increased replication and resolution and frequency of monitoring activities. Those monitoring activities can be repeated and modified, in perpetuity, from archived eDNA samples allowing resource managers to examine ecosystems of the past in support of management needs of the present and future.

Despite these advantages, eDNA also has shortcomings and biases. The tremendous sensitivity of eDNA increases the potential for false positives (Pedersen et al., 2015; Chambert, Miller & Nichols, 2015; Goldberg et al., 2016; Gibbons, Duvallet & Alm, 2018; McKnight et al., 2019; Darling, Jerde & Sepulveda, 2021; Stoeckle et al., 2022). Although false positives can be mitigated through rigorous decontamination approaches including site occupancy modeling, these approaches are not perfect. Our study detected five unexpected fish species in the Port complex with high confidence, all of which are common seafood species processed within the Port’s large-scale active seafood operations. Thus, understanding extraneous sources of exogenous DNA into a given study system (aquaculture, agricultural runoff, aquaria outflow pipes, *etc*.) is important as such sources may influence the interpretation of eDNA metabarcoding results (Yamamoto et al., 2017).

Furthermore, some marine systems are not suitable for eDNA monitoring efforts because of the complex fate and transport or biological and chemical properties of the substrate (Kelly et al., 2014). For example, marine systems with high currents and large tidal exchanges may preclude fine scale comparisons of marine environments given the scale of mixing processes (O’Donnell et al., 2017; Andruszkiewicz et al., 2019). Turbid estuaries can also be difficult to sample because of the impacts of PCR inhibitors and turbidity both acting to limit assay sensitivity (Sanches & Schreier, 2020; Nagarajan et al., 2022). Although field and laboratory methods can alleviate these limitations (Schrader et al., 2012; Polanco et al., 2021a), undoubtedly eDNA metabarcoding may not be suitable to all marine habitats and regions, requiring pilot studies to demonstrate validity of such molecular approaches.

Another limitation of eDNA approaches is the ability to reliably estimate quantitative abundance. Many important management questions involve estimates of abundance, biomass and population demographics (*e.g.*, sex ratio, size frequency), not just presence and absence. However, eDNA researchers are beginning to address these questions (Beng & Corlett, 2020). Recent advances comparing quantitative estimates from eDNA metabarcoding and traditional biomass estimates have found success when applying novel mechanistic frameworks accounting for amplification biases and sampling biases (Kelly, Shelton & Gallego, 2019; McLaren, Willis & Callahan, 2019; Silverman et al., 2020; Silverman et al., 2021) as well as when comparing abundances at ecologically relevant spatial and temporal scales (Tillotson et al., 2018; Yates, Fraser & Derry, 2019; Shelton et al., 2019; Di Muri et al., 2020; Stoeckle et al., 2021). However, given the current uncertainty surrounding quantitative estimates from eDNA metabarcoding approaches, current skepticism of such applications from managers is well warranted (see S1 Supplemental Materials Appendix 3, Fig. S6).

## Conclusions

Given the strengths and limitations of current methods, eDNA metabarcoding approaches are best suited for comprehensive biodiversity inventories in coastal and benthic ecosystems (Meyer et al., 2021). The sensitivity and comprehensiveness of eDNA metabarcoding approaches are particularly useful for biomonitoring efforts where the objective is to determine the location of a given species (Kelly et al., 2014; Bohmann et al., 2014). This includes tracking invasion fronts of marine species of management concern as demonstrated here; as well as identifying species range shifts in response to ocean warming (Beng & Corlett, 2020; Stoeckle, Mishu & Charlop-Powers, 2020; Gold et al., 2021b). Likewise, eDNA metabarcoding is well suited for inventorying endangered and protected species in nearshore and estuary environments (Stoeckle, Soboleva & Charlop-Powers, 2017; West et al., 2020; Larson et al., 2022), although not yet quantitatively. Although the current state of reference databases makes eDNA more suitable for vertebrates than invertebrate taxa, ongoing efforts to create high quality barcode reference datasets for marine invertebrates will resolve this problem (Leray et al., 2019).

Because of the spatial and temporal factors influencing dispersal and degradation of DNA in the marine environment, eDNA metabarcoding is less well suited for tracking highly migratory species across large ocean areas (*e.g.*, mapping Humpback whale or Cuvier’s beaked whales occurrence across the California Current) as the required sampling effort would be prohibitive (Andruszkiewicz et al., 2019; Pinfield et al., 2019; West et al., 2020; Monuki, Barber & Gold, 2021). Likewise, eDNA metabarcoding is not yet appropriate for comparisons of relative abundance (Beng & Corlett, 2020) between environments as is often needed for habitat restoration and no-take zone monitoring efforts (Gold et al., 2021b).

We also highlight the value of the development and application of eDNA metabarcoding in conjunction with marine resource managers as done here (Mosher et al., 2020). Key to the success of this project was a clear outline of the project and monitoring objective, continued and open communication between stakeholders including numerous presentations and workshops to clarify objectives, jargon, and design features, and involvement of resource managers at all phases of the study including design, field collection, laboratory analysis, bioinformatics, interpretation, and writing (Mosher et al., 2020). Although such approaches are not without challenges, such collaborative and integrated efforts dramatically improved the utility and understanding of eDNA metabarcoding for monitoring biodiversity within the Ports of Los Angeles and Long Beach.

Importantly, while there are applications where eDNA can be useful in isolation, it should not be viewed as a wholesale replacement for traditional methods. There will always be intrinsic value in visual surveys and “boots on the ground” surveys, particularly for resource management decisions with substantial costs and consequences (Keller et al., 2022). However, given the power of eDNA, it should be at least be viewed as an important complementary tool to expand the taxonomic and temporal scope of monitoring activities as only eDNA currently provides a cost-effective approach for monitoring important marine ecosystems at high resolution and frequency, critical to understanding how anthropogenic stressors are impacting marine ecosystems during times of unprecedented global change. Therefore, eDNA metabarcoding is well suited to serve as a readily deployable early warning system to inform targeted and consequential management actions (Jerde et al., 2011). Ultimately, with sufficient validation, eDNA methods can provide important complementary biodiversity insights for state and federal biomonitoring efforts required under environmental statutes (NEPA, CEQA, CWA, etc.), improving the capacity and accuracy needed by management agencies to achieve their important ecological mandates.

##  Supplemental Information

10.7717/peerj.14071/supp-1 Supplemental Information 1 eDNA and trawl survey port level species rarefaction curves Species rarefaction curves across sampling stations for trawl surveys (A), eDNA surveys for all observed taxa (B), and eDNA surveys for matching trawl observed taxa. eDNA surveys within a single station ( *n* = 12 bottles) captured over four times the diversity than a single trawl survey. Only two stations ( *n* = 24 bottles) sampled by eDNA surveys were needed to saturate diversity of species observed by trawl surveys.

10.7717/peerj.14071/supp-2 Supplemental Information 2 eDNA and trawl survey port level sample coverage curves Sample coverage curves across sampling stations for trawl surveys (A), eDNA surveys for all observed taxa (B), and eDNA surveys for matching trawl observed taxa. eDNA surveys had higher sample coverage of species detected than trawl surveys, indicating greater taxonomic overlap between replicate bottles than replicate trawls between stations.

10.7717/peerj.14071/supp-3 Supplemental Information 3 eDNA survey station level species rarefaction and sample coverage curves Sample coverage (A) and species rarefaction (B) curves for eDNA surveys at the station level. The average sample coverage estimate across locations within each station was 97.5% (90.5–100%, min–max). These results suggest that an average of 5 (4–19) locations within a station were needed to saturate diversity.

10.7717/peerj.14071/supp-4 Supplemental Information 4 eDNA survey location level sample coverage curves Sample coverage curves for eDNA surveys at the location level with each station plotted separately. The average sample coverage estimate across bottle replicates within each location was 94.0% (82.0%–100%, min–max).

10.7717/peerj.14071/supp-5 Supplemental Information 5 eDNA survey location level species rarefaction curves Species rarefaction curves for eDNA surveys at the location level with stations plotted separately. These results suggest that an average of 7.3 (2–19) bottles within a location were needed to saturate diversity.

10.7717/peerj.14071/supp-6 Supplemental Information 6 eDNA index vs. trawl biomass (kg) eDNA index tracked biomass for three of the five species with sufficient data points to be analyzed. We note that such correlations between eDNA metabarcoding results and visual observations are fraught with challenges as detailed in the Discussion section.

10.7717/peerj.14071/supp-7 Supplemental Information 7 eDNA index vs. trawl abundance (counts) eDNA index tracked biomass for three of the five species with sufficient data points to be analyzed. We note that such correlations between eDNA metabarcoding results and visual observations are fraught with challenges as detailed in the Discussion section.

10.7717/peerj.14071/supp-8 Supplemental Information 8 Compendium of supplemental tables

10.7717/peerj.14071/supp-9 Supplemental Information 9 Supplemental Materials

## Additional Information and Declarations

### DNA Deposition

The following information was supplied regarding the deposition of DNA sequences: The eDNA metabarcoding sequences are available at NCBI SRA: PRJNA851175; SRR19783134 to SRR19783232.

### Data Availability

The following information was supplied regarding data availability: The data is available at Dryad: Gold, Zachary et al. (2022), A manager’s guide to using eDNA metabarcoding in marine ecosystems, Dryad, Dataset, https://doi.org/10.5068/D1B10H. The code is available on GitHub and archived on Zenodo: Zack Gold. (2022). zjgold/Port-of-LA-LB-Project: v1.0.0 (portoflalb). Zenodo. https://doi.org/10.5281/zenodo.6667975.
