# Supplementary figures and images for: A manager’s guide to using eDNA metabarcoding in marine ecosystems
Source: PeerJ. 2022 Nov 15;10:e14071. doi: 10.7717/peerj.14071 (PMC9673773; doi:10.7717/peerj.14071)

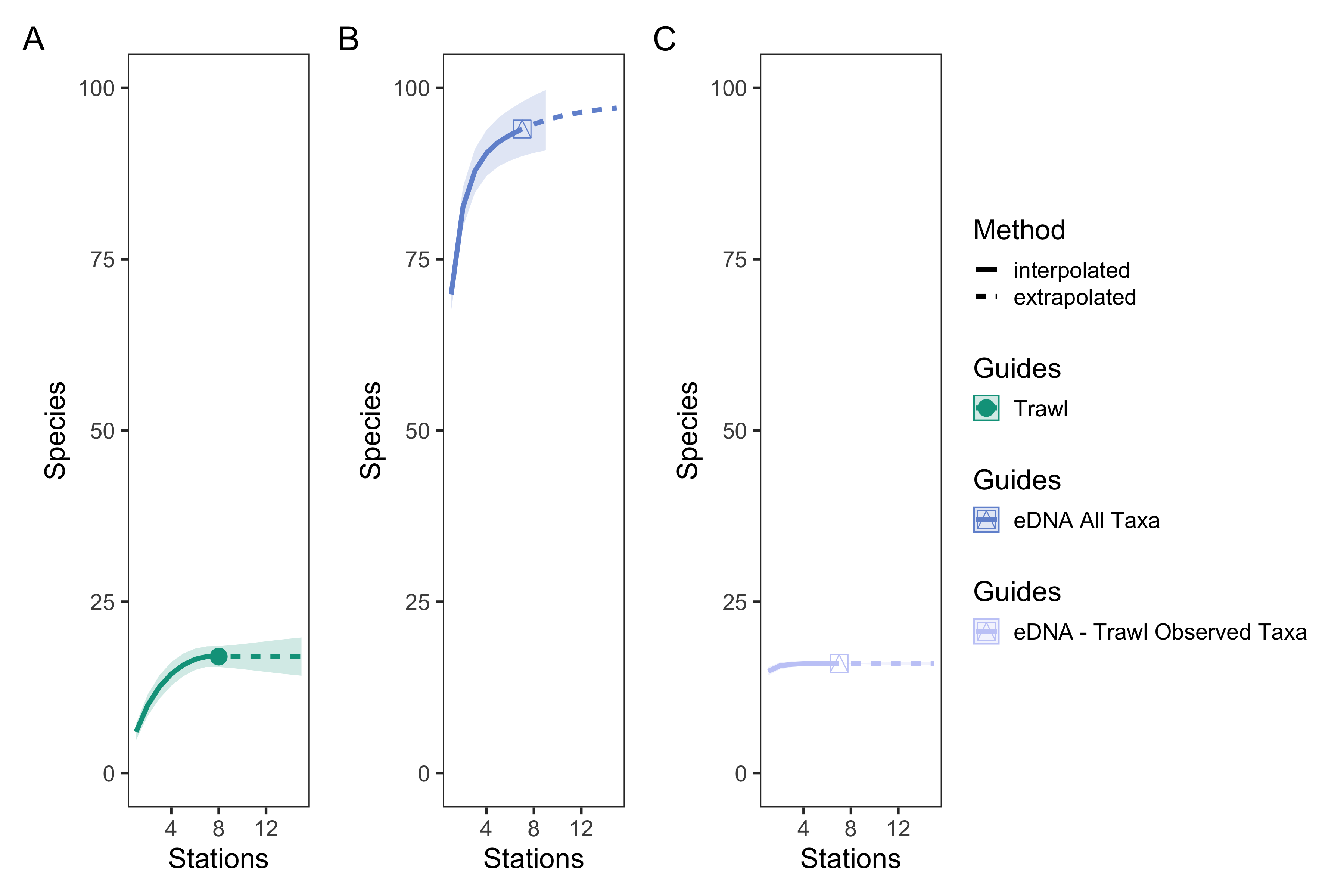

Supplement: Supplemental Information 1 — Species rarefaction curves across sampling stations for trawl surveys (A), eDNA surveys for all observed taxa (B), and eDNA surveys for matching trawl observed taxa. eDNA surveys within a single station ( n = 12 bottles) captured over four times the diversity than a single trawl survey. Only two stations ( n = 24 bottles) sampled by eDNA surveys were needed to saturate diversity of species observed by trawl surveys. [file peerj-10-14071-s001.png]

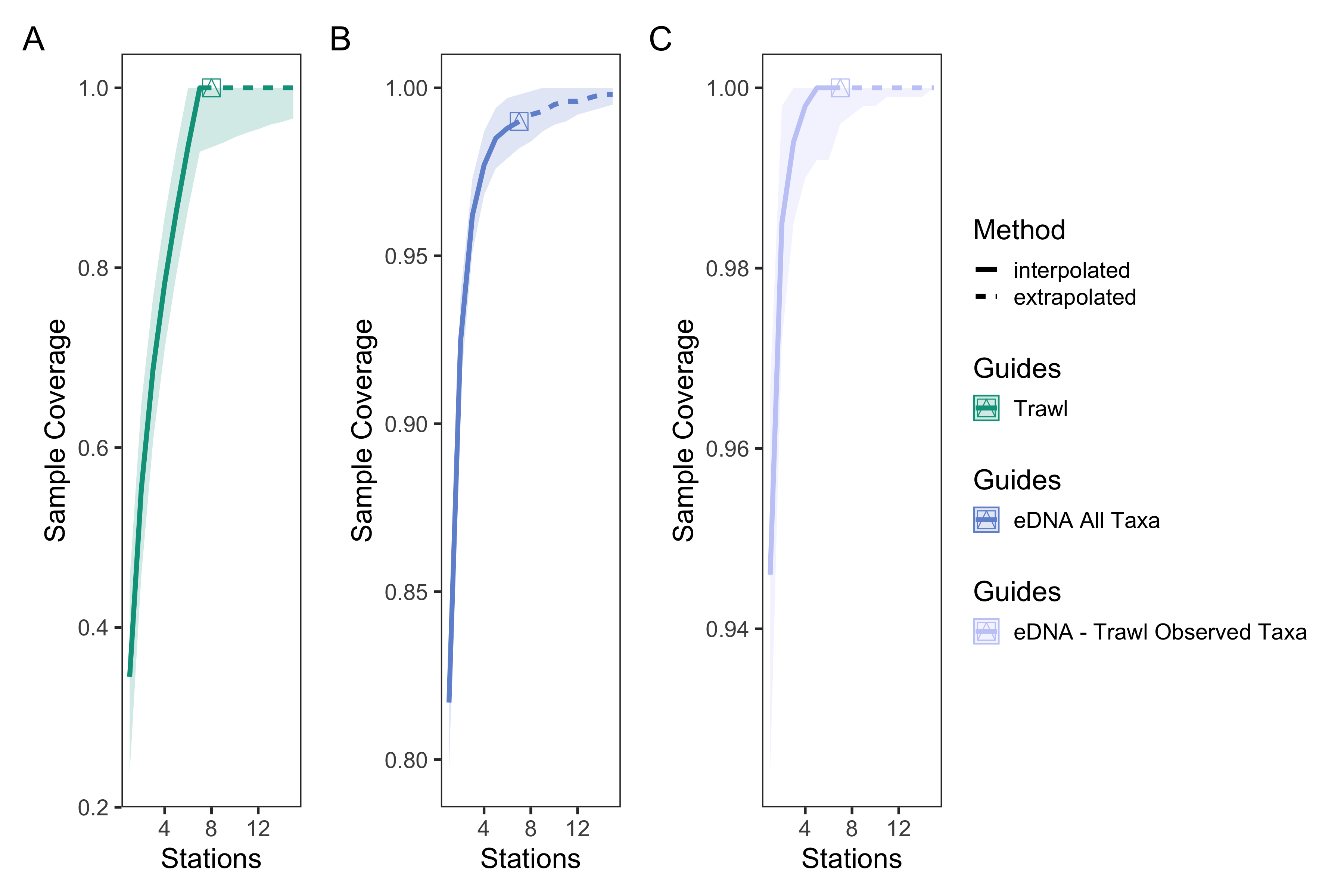

Supplement: Supplemental Information 2 — Sample coverage curves across sampling stations for trawl surveys (A), eDNA surveys for all observed taxa (B), and eDNA surveys for matching trawl observed taxa. eDNA surveys had higher sample coverage of species detected than trawl surveys, indicating greater taxonomic overlap between replicate bottles than replicate trawls between stations. [file peerj-10-14071-s002.png]

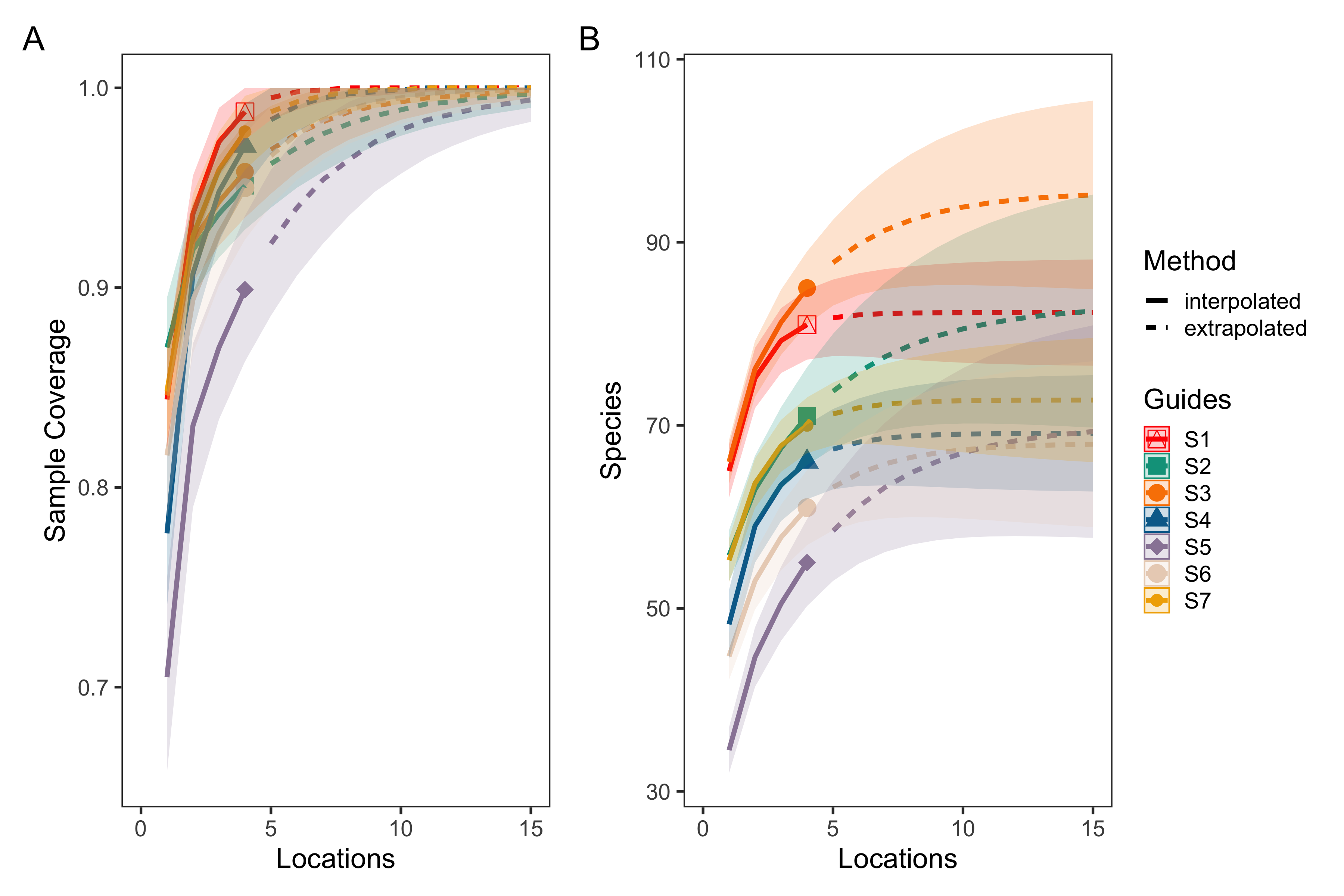

Supplement: Supplemental Information 3 — Sample coverage (A) and species rarefaction (B) curves for eDNA surveys at the station level. The average sample coverage estimate across locations within each station was 97.5% (90.5–100%, min–max). These results suggest that an average of 5 (4–19) locations within a station were needed to saturate diversity. [file peerj-10-14071-s003.png]

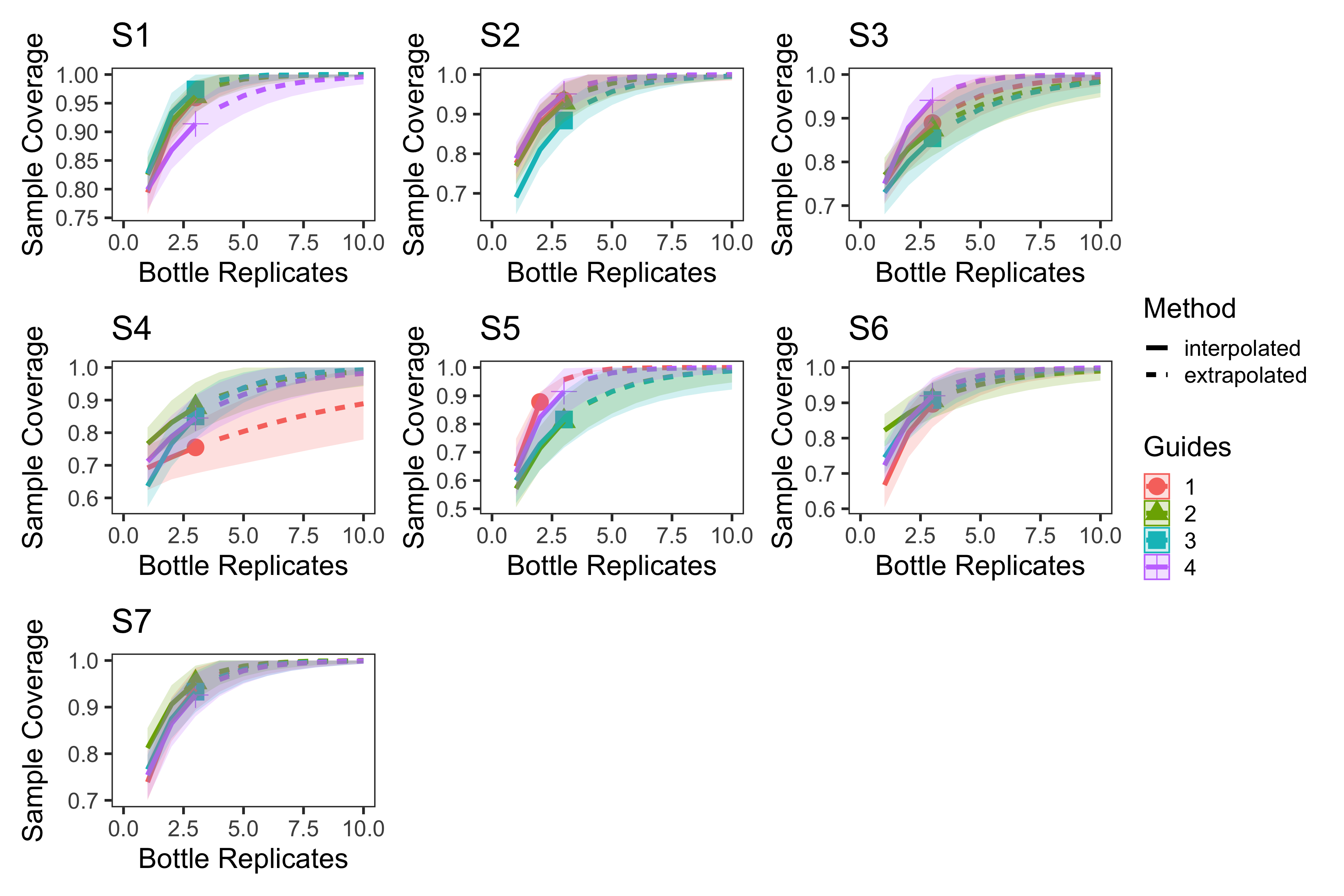

Supplement: Supplemental Information 4 — Sample coverage curves for eDNA surveys at the location level with each station plotted separately. The average sample coverage estimate across bottle replicates within each location was 94.0% (82.0%–100%, min–max). [file peerj-10-14071-s004.png]

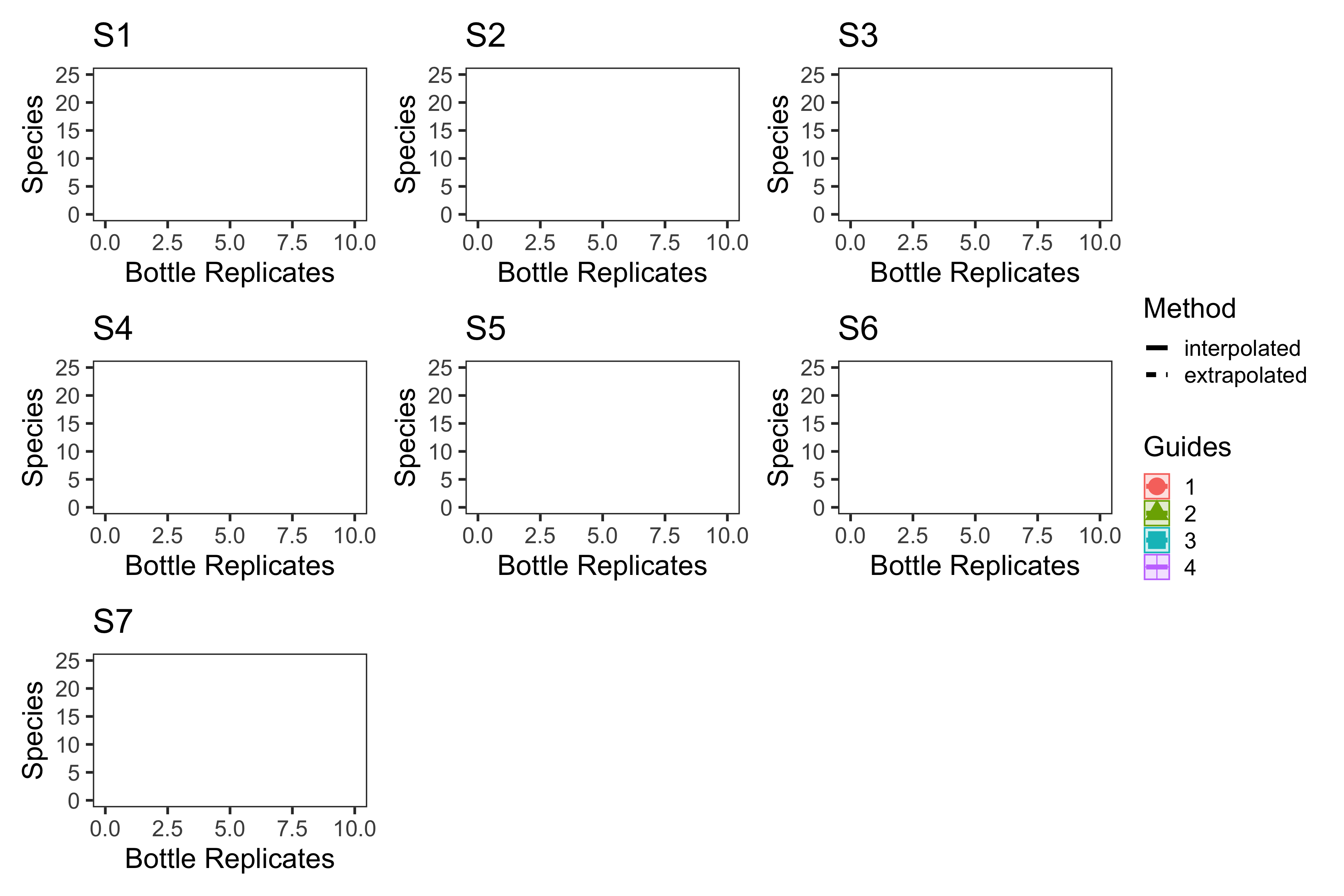

Supplement: Supplemental Information 5 — Species rarefaction curves for eDNA surveys at the location level with stations plotted separately. These results suggest that an average of 7.3 (2–19) bottles within a location were needed to saturate diversity. [file peerj-10-14071-s005.png]

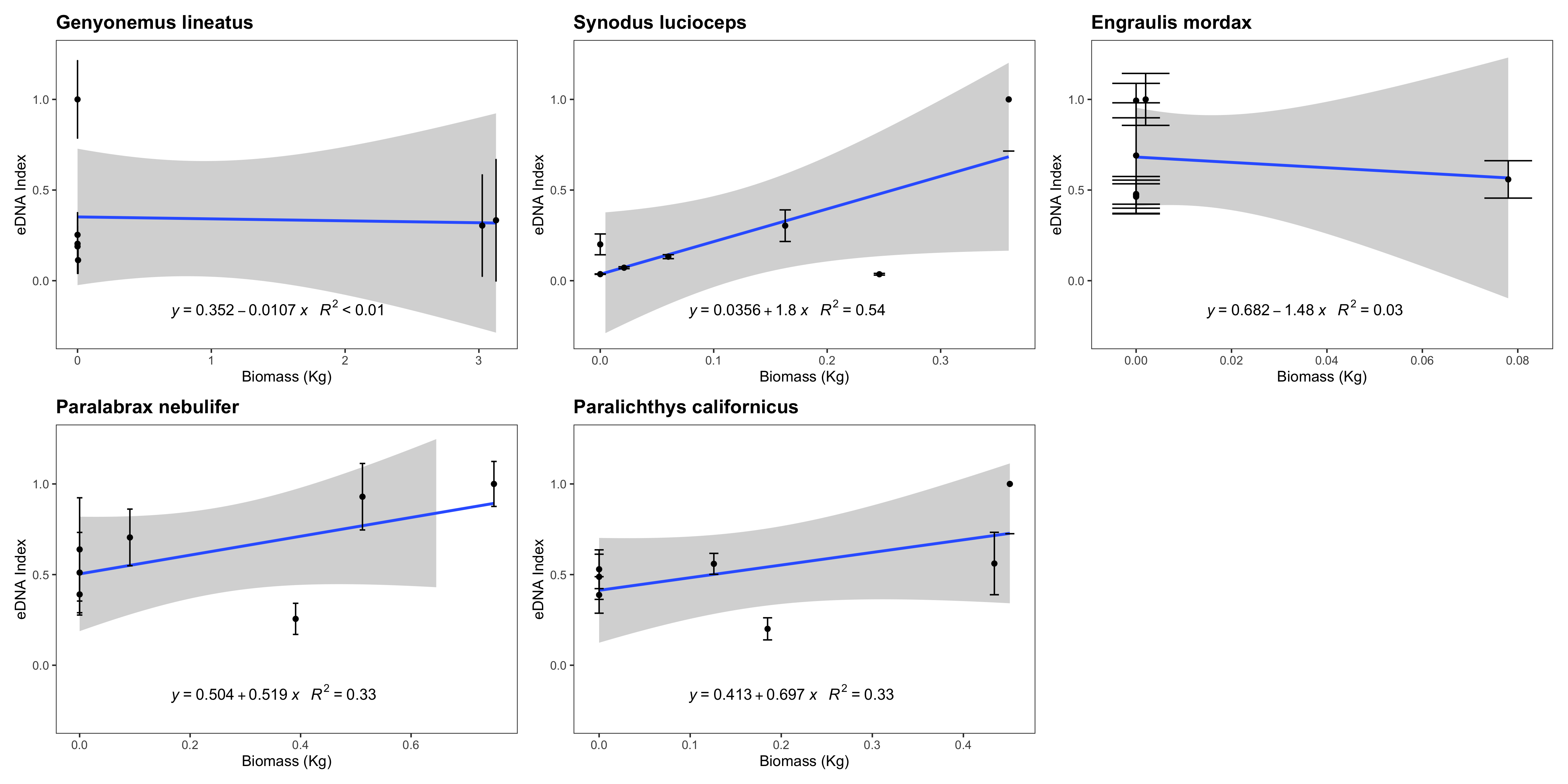

Supplement: Supplemental Information 6 — eDNA index tracked biomass for three of the five species with sufficient data points to be analyzed. We note that such correlations between eDNA metabarcoding results and visual observations are fraught with challenges as detailed in the Discussion section. [file peerj-10-14071-s006.png]

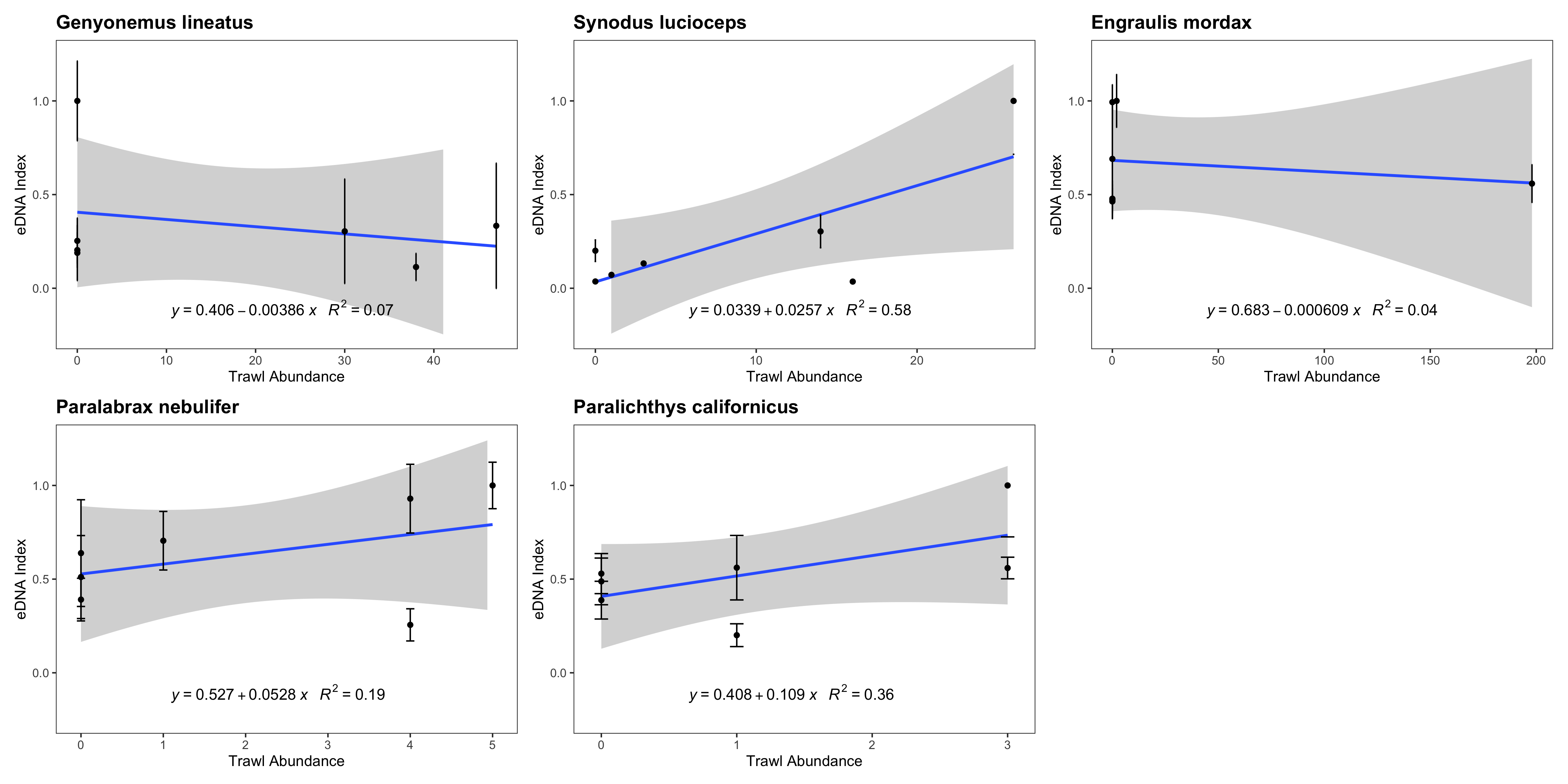

Supplement: Supplemental Information 7 — eDNA index tracked biomass for three of the five species with sufficient data points to be analyzed. We note that such correlations between eDNA metabarcoding results and visual observations are fraught with challenges as detailed in the Discussion section. [file peerj-10-14071-s007.png]
