# Supplementary material for: A manager’s guide to using eDNA metabarcoding in marine ecosystems
Source: PeerJ. 2022 Nov 15;10:e14071. doi: 10.7717/peerj.14071 (PMC9673773; doi:10.7717/peerj.14071)
Supplement: Supplemental Information 9 [file peerj-10-14071-s009.docx]

***A Manager’s Guide to Using eDNA Metabarcoding in Marine Ecosystems***

**Guidance – remove this box before submitting!**

Yellow callout boxes provide general notes. Please remove the yellow boxes before submitting. For full guidance see <https://peerj.com/about/author-instructions>

Blue highlighted example text should be replaced or removed with your own information.

| **DO**  –Use clear and grammatically correct English.  –Save as US Letter size format.  –Ensure line numbering is enabled.  –Align text LEFT.  –Ensure title, abstract, and author information matches what is entered online during submission. | **DO NOT**  –Embed ANY figures or tables in the text. Instead, upload a separate file for each on the file uploads page when submitting. Example – If you have 3 figures, then you will upload 3 figure files & be asked to add a figure title for each. See <https://peerj.com/about/author-instructions/#figures> for figure formats. |
| --- | --- |

Zachary Gold^1^, Adam R. Wall^2^, Teia M. Schweizer^3^, N. Dean Pentcheff^2^, Emily E. Curd^4^, Paul H. Barber^1^, Rachel S. Meyer^1,5^, Robert Wayne^1^, Kevin Stolzenbach^6^, Kat Prickett^7^, Justin Luedy^8^, Regina Wetzer^2^

^1^Department of Ecology and Evolutionary Biology, University of California Los Angeles, Los Angeles, CA, USA

^2^ Diversity Initiative for the Southern California Ocean (DISCO), Natural History Museum of Los Angeles County, Los Angeles, CA, USA

^3^Department of Fish and Wildlife Conservation Biology, Colorado State University, Fort Collins, CO, USA

^4^ Department of Natural Sciences, Landmark College, Putney, VT, USA

^5^Department of Ecology and Evolutionary Biology, University of California Los Angeles, Los Angeles, CA, USA

^6^Wood Environment and Infrastructure, Inc., San Diego, CA, USA

^7^Port of Los Angeles, San Pedro, CA, USA

^8^Port of Long Beach, Long

**Supplemental Materials**

**Table of Contents**

- S1 Appendix I: Full Methods
- S1 Appendix II: Supplemental Results
- S1 Appendix III: Biomass Comparisons
- Supplemental Figures and Tables
  - Figure S1. eDNA and Trawl Survey Port Level Species Rarefaction Curves
  - Figure S2. eDNA and Trawl Survey Port Level Sample Coverage Curves
  - Figure S3. eDNA Survey Station Level Species Rarefaction and Sample Coverage Curves
  - Figure S4. eDNA Survey Location Level Sample Coverage Curves
  - Figure S5. eDNA Survey Location Level Species Rarefaction Curves
  - Figure S6. eDNA Index vs. Trawl Biomass (kg)
  - Figure S7. eDNA Index vs. Trawl Abundance (counts)
- Supplemental Tables
  - Table S1. eDNA Sample Metadata
  - Table S2. Trawl Sampling Metadata
  - Table S3. Trawl Abundances (counts) data
  - Table S4. Trawl Biomass (kg) data
  - Table S5. MiFish 12S Universal Teleost Sequence Read Counts Prior to Site Occupancy Modeling Assigned with Gold et al. 2021 Curated Reference Database
  - Table S6. MiFish 12S Elasmobranch Sequence Read Counts Prior to Site Occupancy Modeling Assigned with Gold et al. 2021 Curated Reference Database
  - Table S7. MiFish 12S Universal Teleost Sequence Read Counts Prior to Site Occupancy Modeling Assigned with Curd et al. 2019 12S database
  - Table S8. MiFish 12S Elasmobranch Sequence Read Counts Prior to Site Occupancy Modeling Assigned with Curd et al. 2019 12S database
  - Table S9. CO1 Sequence Read Counts Prior to Site Occupancy Modeling
  - Table S10. 16S Sequence Read Counts Prior to Site Occupancy Modeling
  - Table S11. Cal-NEMO Invasive Species List
  - Table S12. MiFish 12S Universal Teleost Sequence Read Counts After Site Occupancy Modeling
  - Table S13. MiFish 12S Elasmobranch Sequence Read Counts After Site Occupancy Modeling
  - Table S14. CO1 Sequence Read Counts After Site Occupancy Modeling
  - Table S15. 16S Sequence Read Counts After Site Occupancy Modeling
  - Table S16. Combined 12S Sequence Read Counts After Site Occupancy Modeling
  - Table S17. Combined 12S eDNA Index Scores After Site Occupancy Modeling
  - S18. PERMANOVA and Betadisper Results
  - Table S19. Port-wide Rarefaction Results
  - Table S20. Station Level Rarefaction Results
  - Table S21. Location Level Rarefaction Results

# S1. APPENDIX I: FULL METHODS

## Contamination precautions

False positives may result from contamination during eDNA collection as well as during DNA extraction and amplification. To prevent contamination in both field sampling and lab work, we followed precautions outlined in Goldberg et al., 2016. Specifically, personnel wore nitrile gloves at all times during field and lab sample processing. Prior to eDNA sample collection, we sterilized all containers, supplies, and work surfaces with 10% bleach solution followed by 30 min ultraviolet light (UV) treatment. We conducted all eDNA filter extractions and PCR preparations in an AirClean 600 PCR Workstation (Creedmoor, NC, USA) located in a clean room dedicated to DNA extractions and PCR preparations at the University of California, Los Angeles (UCLA). Before and after use, we cleaned the AirClean 600 PCR Work-station and pipettes with 10–30% bleach followed by a 30 min UV treatment. We used filtered pipette tips for all pre- and post-PCR procedures. These and other consumables were decontaminated by a 30 min UV treatment. In addition to the above, we included negative controls at all sample processing steps including field blanks, extraction blanks, and PCR blanks, we also used two positive controls to test for index hopping.

## eDNA sample collection

We first sampled water for eDNA prior to trawling to ensure that trawling did not influence eDNA signals. To standardize the collection of seawater, we used three 5 L Niskin bottles (General Oceanics, Miami, FL, USA), with an anchor set to capture water from approximately 2 m above the seafloor (See Appendix S1 for full method details). Upon arrival at each location, sampling bottles were flushed continuously with surface water for a minimum of five minutes before deployment. Sea water from each sampling bottle was transferred to a sterile one L Kangaroo Enteral Feeding Gravity Bag (Covidien PLC, Dublin, Ireland). The first 0.5 L of each sample was used to rinse the bag and then discarded, the bag was then filled with one L of sample water and gravity filtered through a 0.22 μm Sterivex filter (Millipore Corp. Burlington, MA, USA) (Miya et al., 2015; Port et al., 2016; Curd et al., 2019). Nalgene tubing and connectors were sterilized prior to assembly in the AirClean PCR hood. Filtration was carried out in the ship cabin, shielded from sunlight, for 40 minutes or until one L of sample was filtered, whichever came first (Table S1). Previous testing had demonstrated that after 40 minutes a filter was clogged and longer filtration was fruitless (data not shown). Filter cartridges were then placed into individual sterile Whirl-Pak bags (Nasco, Fort Atkinson, WI, USA) and then placed in a cooler with dry ice (Curd et al., 2019). The resulting samples were kept on ice for up to 6 hours before being transferred to a -20° F freezer. As a negative control, we processed a field blank at each station that consisted of one L of UltraPure DNAse/RNAse Free Distilled Water (Invitrogen, Carlsbad, CA) following standard methods (Goldberg et al., 2016; Kelly, Gallego & Jacobs-Palme, 2018).

## Environmental DNA extractions, amplification and sequencing

At UCLA, all eDNA filter extractions and PCR preparations were conducted in an AirClean 600 PCR Work-station (Creedmoor, NC, USA) in a clean room dedicated to DNA extractions. The AirClean 600 PCR Work-station and pipettes were decontaminated before and after use with 10% bleach, 70% ethanol, and DNA Away, followed by a 20 min ultraviolet light (UV) treatment. Filtered pipette tips were used for all protocols. There were negative controls (extraction blanks and PCR blanks) for all steps in the sample processing.

Sterivex filters were extracted with a DNAeasy Tissue and Blood Qiagen Kit (Spens et al., 2017) protocol. Proteinase K and ATL buffer was added to the filter cartridge and incubated overnight at 56° C. DNA was amplified with primer sets with Illumina Nextera adapter modifications for use with Nextera XT indexes (Product Number FC-131-2001 through -2004; 5200 Illumina Way, San Diego, CA 92122). The primer sets presented here were: (1) MiFish Universal Teleost 12S primer (176bp) (Miya et al., 2015); (2) MiFish Universal Elasmobranch 12S primer (186bp) (Miya et al., 2015). Samples were also amplified for (3) metazoan COI primers (313bp) (Meyer et al., 2021); and (4) metazoan *16S* primers (~115bp) (Kelly et al., 2016), data not shown. PCR amplifications in triplicate for each primer pair contained 12.5 μL QIAGEN Multiplex Taq PCR 2x Master Mix, 6.5 µL dH2O, 2.5 µL of each primer (2 µmol/L), and 1 μL DNA template (25 µL total reaction volume). PCR thermocycling employed a touchdown profile with an initial denaturation at 95° C for 15 min to activate the DNA polymerase followed by 13 cycles with a denaturation step at 94° C for 30 sec, an annealing step with temperature starting at 69.5° C for 30 sec (temperature was decreased by 1.5° C every cycle until 50° C was reached), and an extension step at 72° C for 1 min. An additional 35 cycles were then carried out at an annealing temperature of 50° C using the same denaturation and extension steps above, followed by a final extension at 72° C for 10 min. All PCRs included a negative control, where molecular grade water replaced the DNA template. All PCR products were run on 2% agarose gels to ensure amplification success and correct product size.

To prepare PCR products for sequencing, we pooled triplicate PCR reactions using 5 µL volume from each PCR. Pooled samples were cleaned using Serapure magnetic beads (Faircloth & Glenn, 2014), and cleaned PCR product concentrations were quantified using the high sensitivity Quant-iT™ dsDNA Assay Kit (Thermofisher Scientific, Waltham, MA, USA) on a Victor3™ plate reader (Perkin Elmer Waltham, MA, USA). Sample DNA libraries were prepared using the Nextera Index Kits (Illumina, San Diego, CA, UCA) and KAPA HiFi HotStart Ready Mix (Kapa Biosystems, Wilmington, MA, USA). This second indexing PCR was performed using a 25 μL reaction mixture containing 12.5 μL of Kapa HiFi Hotstart Ready mix, 0.625 μL of primer i7, 0.625 μL of primer i5, and 10 ng of template DNA, and used the following thermocycling parameters: denaturation at 95° C for 5 min, 5 cycles of denaturation at 98° C for 20 sec, annealing at 56° C for 30 sec, extension at 72° C for 3 min, followed by a final extension at 72° C for 5 min. All indexed PCR products were run on 2% agarose gels to ensure successful PCR and correct product size. Resulting libraries were bead cleaned and quantified as described above. Finally, we pooled indexed libraries by barcode in equimolar concentration, and then sequenced each of the four libraries on a MiSeq at the Technology Center for Genomics & Bioinformatics (University of California Los Angeles, CA, USA), using Reagent Kit V3 with 20% PhiX added to all sequencing runs.

## Bioinformatic analysis and taxonomic determination

Taxonomic assignments of eDNA metabarcoding reads require a comprehensive sequence reference library. To generate these libraries for our given marker sets, we used *CRUX* (Constructing Reference libraries Using eXisting tools) (Curd et al., 2019) to query NCBI GenBank October 2019 for all available sequences that overlapped with each of our primer regions. One library was constructed for both the MiFish Teleost and Universal *12S* primer set given the locus similarity, one library was constructed for the *CO1* metazoan primer as detailed, and one library was constructed for the *16S* metazoan primer set using the default *CRUX* parameters (Curd et al., 2019).

We used the *Anacapa Toolkit* (Curd et al., 2019) for amplicon sequence variant parsing, taxonomic assignment, and quality control. The quality control step of the Anacapa Toolkit trims extraneous adapter sequences used to identify each unique sample, removes low quality reads, and sorts reads by metabarcode primer sequence. A key advantage of the *Anacapa Toolkit* is that it can simultaneously process raw fastq reads for samples with single or multiple metabarcode targets generated on Illumina MiSeq machines. It is also not required that all samples contain reads for each metabarcode, thus allowing users to combine multiple projects or targets on the same sequencing run while only running the pipeline once.

The amplicon sequence variant (ASV) parsing step uses DADA2 (Callahan et al., 2016) to dereplicate our metabarcodes. Unlike OTUs, which cluster sequences using an arbitrary sequence similarity (e.g. 97%, which corresponds to a gross average genetic sequence difference across all of life), ASVs are unique sequence reads determined using Bayesian probabilities of known sequencing error. These unique sequences can differ by as little as two base pairs, providing improved taxonomic resolution and an increase in observed diversity (Callahan et al., 2016; Amir et al., 2017).

Next the Anacapa toolkit module assigns taxonomy to ASVs using Bowtie 2 (Langmead & Salzberg, 2012) and a Bowtie 2-specific Bayesian Least Common Ancestor (BLCA) algorithm. All ASVs are first globally aligned against the CRUX database using Bowtie 2. Any ASV that fails to align is then aligned locally. The best hits (the top 100 Bowtie 2 returns) are then processed with the BLCA script to assign taxonomy. The Bowtie 2 BLCA algorithm was adapted from https://github.com/qunfengdong/BLCA. BLCA uses pairwise sequence alignment to calculate sequence similarity between query sequences (a given ASV sequence found in the environment) and reference library hits. Taxonomy is assigned based on the lowest common ancestor of multiple reference library hits for each query sequence. The reliability of each taxonomic assignment is then evaluated through bootstrap confidence scores (Gao et al., 2017). Scores are based on Bayesian posterior probability which quantify the similarity of reference barcode sequences to the query sequence. The higher the similarity between the database barcode sequence and the query sequence, the greater the contribution to the taxonomic assignment of the query. Ultimately, this method provides a strong probabilistic basis for evaluating taxonomic assignments with bootstrap confidence scores.

For the two fish primer sets, taxonomic assignment was conducted following benchmarking by Gold et al. (2021) using a taxonomic cutoff score of 60 and minimum alignment of 80%. Two different combinations of reference databases were used for taxonomic assignment. We first assigned taxonomy only using the *CRUX* October 2019 reference database which lacked region specific fish barcodes here in referred to as the limited database. We then assigned taxonomy using a combination of the regional and global reference databases which contained 252 additional California Current species described in Gold et al. 2021 here in reference to as the comprehensive database. For the comprehensive database assignments, taxonomy was first assigned using the curated regional database of California Current Large Marine Ecosystem fishes to identify native taxa. We then re-assigned taxonomy using the global *CRUX* generated database to identify non-native and non-fish species. Taxonomic assignments of ASVs were synonymized between both methods by prioritizing higher resolution assignments (i.e. species level vs. genus level).

As compared to the *12S* MiFish primer sets, the two universal primer sets *CO1* and *16S* have substantially less complete reference databases, and they also lack comprehensive marker validation and benchmarking, thus reducing the accuracy of taxonomic assignments (Edgar, 2018; Curd et al., 2019). Thus to minimize both misclassification and overclassification of *CO1* and *16S* sequences we used a conservative taxonomic cutoff score of 80 and minimum sequence alignment of 95%.

## Bioinformatic decontamination

After processing the raw sequence reads through the *Anacapa Toolkit*, the resulting species community tables were transferred into R (R Core Team 2016) for subsequent decontamination and downstream data analysis using *phyloseq* (version: 1.32.0) (McMurdie & Holmes, 2013) and *ranacapa* (version 1.0) (Kandlikar et al., 2018) *R* packages. The raw species community table needs to be decontaminated to eliminate potential field contamination, lab contamination, and sequence index hopping (Goldberg et al., 2016; Costello et al., 2018). Field and lab contamination can arise because of inadequate sterile procedures, careless laboratory work, and reagents, particularly enzymes which are generated from living organisms (Goldberg et al., 2016). Sequence index hopping occurs when the DNA index tag used to label each unique sample chemically swaps with the DNA index tag of another sample, leading to cross contamination of species between samples. However, to address these sources of contamination, we followed rigorous sterile procedures and sequenced our libraries on the MiSeq platform that has less index hopping compared to Illumina sequencers that use patterned flow cells.

In addition to these precautionary steps, we also implemented a decontamination procedure that eliminates any remaining sources of contamination (Kelly, Gallego & Jacobs-Palme, 2018 [supplemental methods], Kelly, Shelton & Gallego, 2019 [supplemental methods], Gallego et al., 2020 [supplemental methods]). One approach for decontamination is to subtract reads based on the prevalence of ASVs in negative control (McKnight et al., 2019). However, index hopping or cross-contamination can also result in the presence of abundant ASVs from true samples in negative controls (Costello et al., 2018), which risks removal of the most abundant and prevalent ASVs if strict removal decontamination is practiced. Therefore we applied the site occupancy modeling approach of Kelly et al. 2018 to retain only ASVs that occurred in high prevalence across locations and stations. We did not subtract reads based on negative controls given the occurrence of ASVs with the highest observed read counts. Following Kelly et al. 2018, we also excluded samples from analyses that had low read depth.

## Normalizing eDNA reads across samples

Relating the number of sequence reads in a sample to the number of individuals of a taxon is extremely challenging. Not only do different organisms shed DNA at different rates, but the process of PCR amplification is known to have sequence-specific biases (Kelly, Shelton & Gallego, 2019; McLaren, Willis & Callahan, 2019; Silverman et al., 2021). However, it is possible to address some of those known biases in ways that make it plausible to explore eDNA’s ability to yield insights into relative abundance estimates. This metric assumes that PCR biases originate from template-primer interactions that remain constant across eDNA samples and align well with recent empirical and theoretical work (Shelton et al., 2016; Kelly, Shelton & Gallego, 2019; McLaren, Willis & Callahan, 2019; Silverman et al., 2021). Intuitively, therefore, it should be possible to infer information about taxon abundance by using appropriate normalizations of the counts of reads in each sample.

Thus we transformed our data into an “eDNA index” following Kelly et al. 2019. The eDNA index transformation is conducted by first normalizing all reads for a particular sequence by the total number of reads in each sample, then scaling those proportions to the largest observed proportion for that sequence across all samples. This results in a sequence-specific (species-specific) scaling between 0 to 1, where 1 is the sample with the highest number of reads for a given species and 0 is the least.

## Spatial Analyses

We conducted species rarefaction analysis at the level of the Port, station, and location to compare sample coverage estimates (for a given set of sampling units, what fraction of total species were discovered) using the iNext package (Hsieh, Ma & Chao, 2016). We then calculated pairwise similarity between eDNA samples using Bray-Curtis similarity on eDNA index scores using the *vegan* package. This was followed by employing a PERMANOVA and Betadisp analysis using the *vegan* package to calculate the apportioned variation between the pairwise similarity of each sample against site, station, date collected, and sample volume filtered, visualizing the results using a principal coordinates analysis (PCoA) ordination using the *phyloseq* and *vegan* packages.

# S1. APPENDIX II: Supplemental Results

From the 84 eDNA samples collected we generated a total of 14.9 million raw reads for the two MiFish primer sets. After processing via the *Anacapa Toolkit* and decontamination, we retained 705 ASVs across 83 samples and 7.1 million reads for the MiFish Teleost primer set, and 569 ASVs across 83 samples and 3.2 million reads for the MiFish Elasmobranch primer set. Sample read depth ranged from 8,950 to 222,481 reads (mean 62,118 reads).

We generated a total of 7.7 million raw reads for the *CO1* dataset and 8.5 million raw reads for the *16S* dataset. After processing via the *Anacapa Toolkit* and decontamination, we retained 2,791 ASVs across 79 samples and 3.2 million reads for the Leray *CO1* primer set, and 888 ASVs across 77 samples and 6.9 million reads for the Kelly *16S* primer set. Sample read depth ranged from 11,292 to 94,804 reads (mean 40,526 reads) for the *CO1* dataset and from 8,078 to 173,953 reads (mean 90,451 reads) for the *16S* dataset.

*Invasive species identified*

Invasive species identified by eDNA include the Asian mussel (*Musculista senhousia*), Devil weed (*Sargassum horneri*), Scaly tunicate (*Microcosmus squamiger*), Brown bryozoan (*Bugula neritina*), Stalked seaquirt (*Styela clava*), Asian semele (*Theora lubrica*), Ambiguous bryozoan (*Anguinella palmata*), Red encrusting bryozoan (*Cryptosula pallasiana*), Gray compound tunicate (*Diplosoma listerianum*), Carpet sea squirt (*Didemnum vexillum*), Japanese skeleton shrimp (*Caprella mutica*), and Chain tunicate (*Botrylloides violaceus*) (Table S9 & S10).

*Seafood target species identified*

Seafood target species with high probability of occurrence included [Mahi-mahi (*Coryphaena hippurus*), Chinook salmon (*Oncorhynchus tshawytscha*), Skipjack tuna (*Katsuwonus pelamis*), Eastern Pacific bonito (*Sarda chiliensis*), and Albacore tuna (*Thunnus alalunga*)] (Table S16).

*Spatial structuring of eDNA*

We found a significant difference in dispersion of vertebrate communities between stations, largely driven by the high variance at S3 and S5 (betadisper, p< 0.001 Table S18). We found no difference in dispersion between locations within stations (betadisper, p>0.05 Table S18).

eDNA surveys had higher rates of sample coverage and greater rates of species saturation than trawl surveys (Figure S1-5).


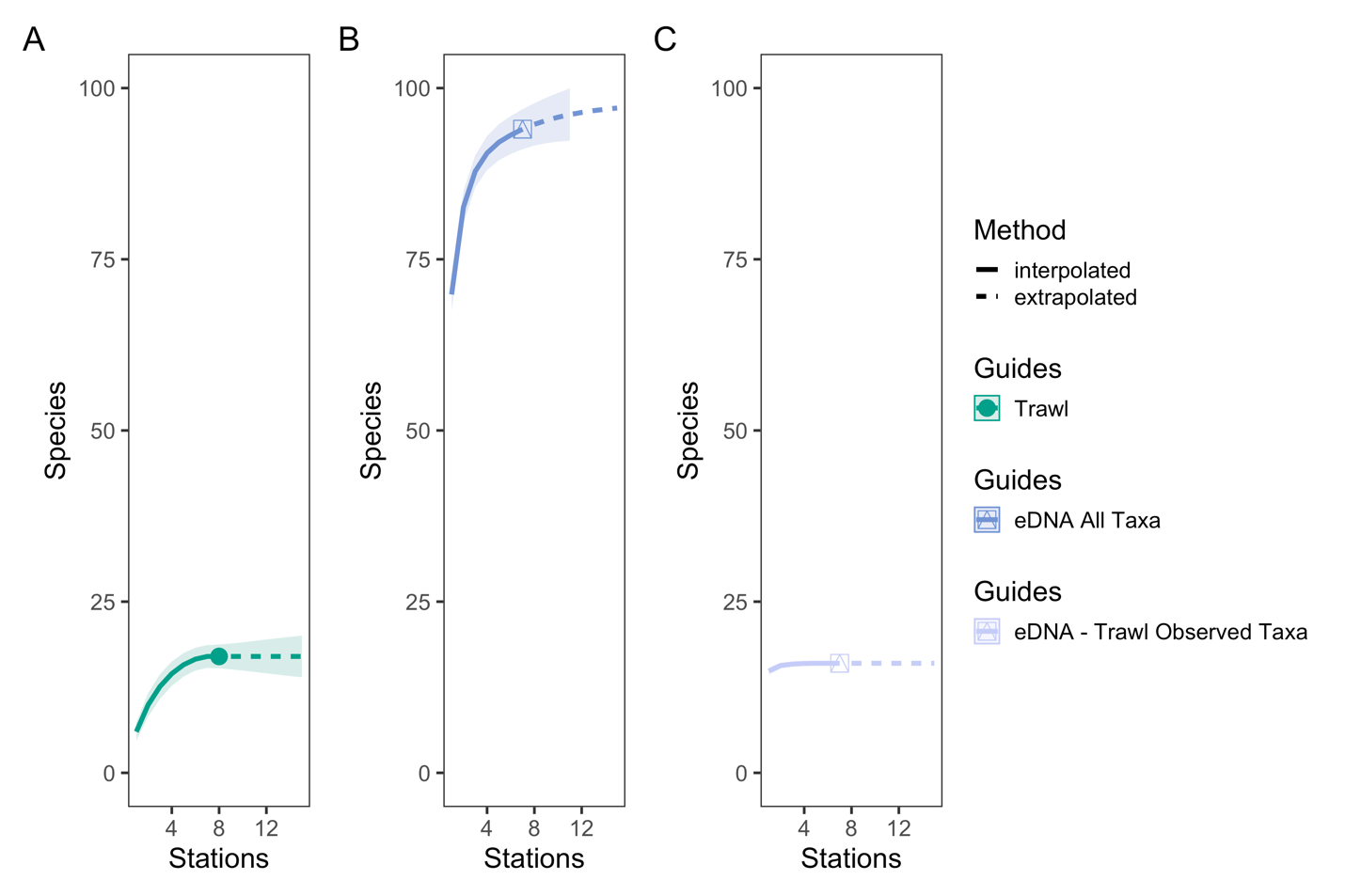


**Figure S1. eDNA and Trawl Survey Port Level Species Rarefaction Curves**

Species rarefaction curves across sampling stations for trawl surveys (A), eDNA surveys for all observed taxa (B), and eDNA surveys for matching trawl observed taxa. eDNA surveys within a single station (n=12 bottles) captured over four times the diversity than a single trawl survey. Only two stations (n=24 bottles) sampled by eDNA surveys were needed to saturate diversity of species observed by trawl surveys.

**
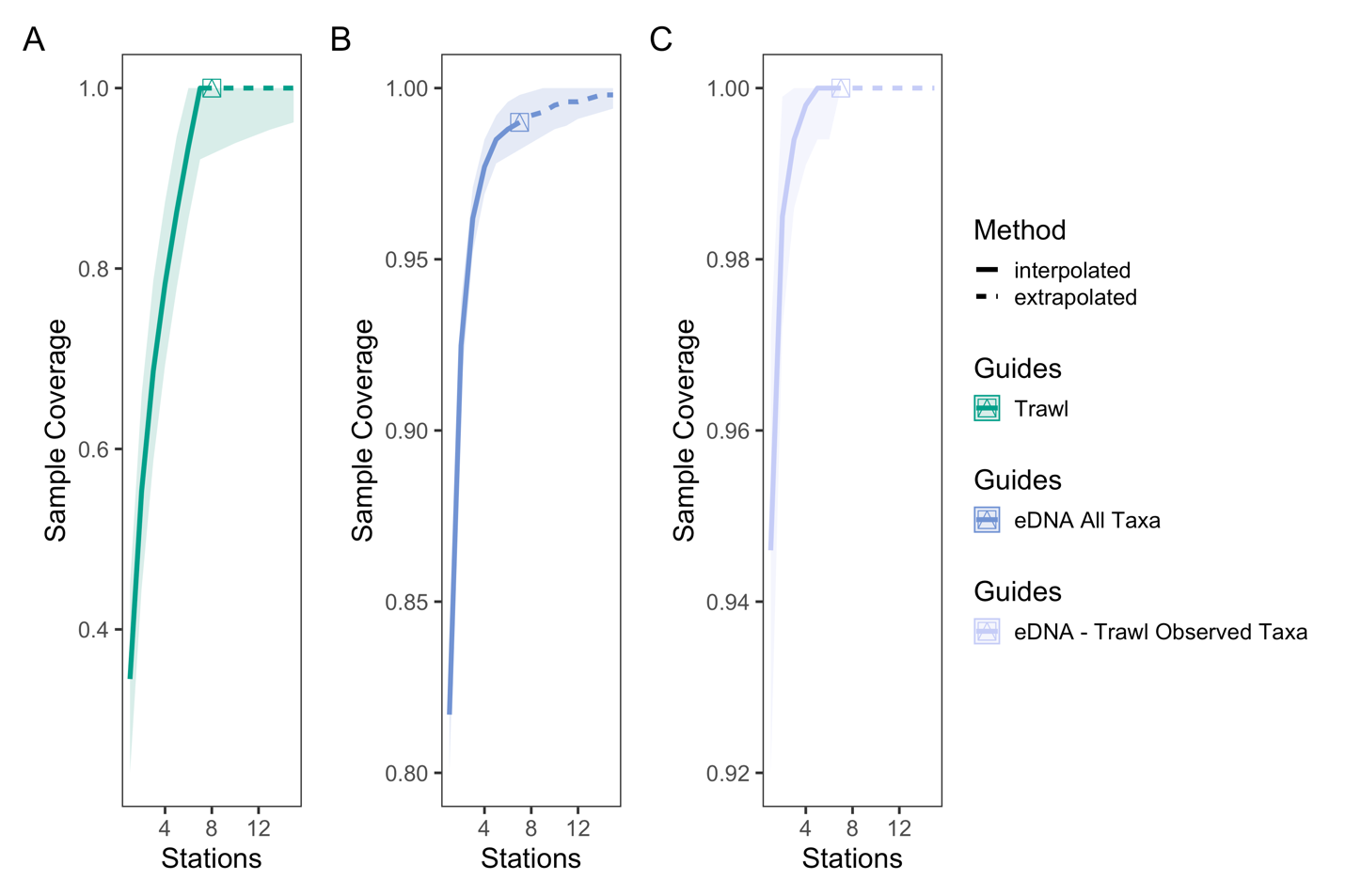
**

**Figure S2. eDNA and Trawl Survey Port Level Sample Coverage Curves**

Sample coverage curves across sampling stations for trawl surveys (A), eDNA surveys for all observed taxa (B), and eDNA surveys for matching trawl observed taxa. eDNA surveys had higher sample coverage of species detected than trawl surveys, indicating greater taxonomic overlap between replicate bottles than replicate trawls between stations.

**
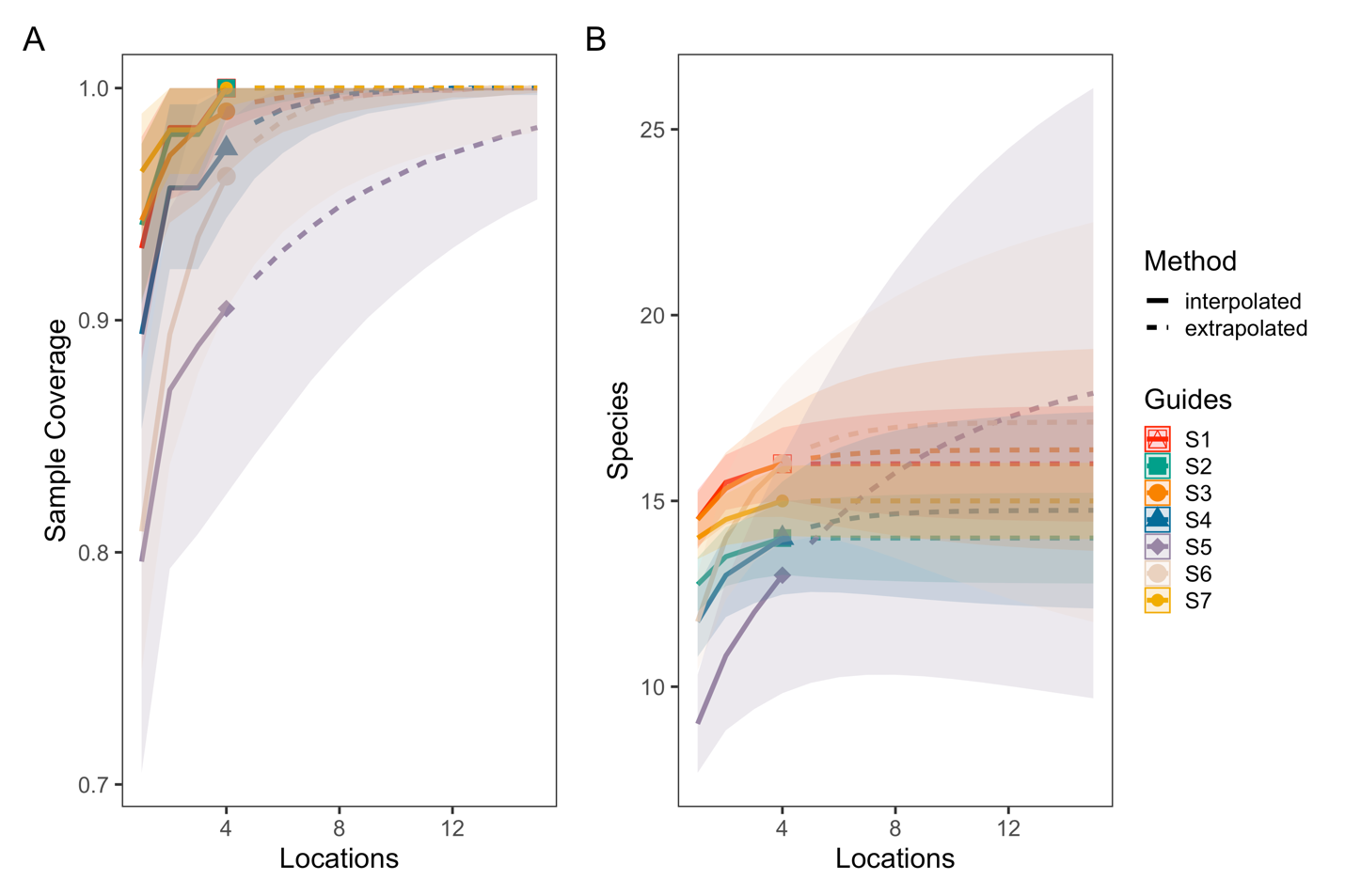
**

**Figure S3. eDNA Survey Station Level Species Rarefaction and Sample Coverage Curves**

Sample coverage (A) and species rarefaction (B) curves for eDNA surveys at the station level. The average sample coverage estimate across locations within each station was 97.5% (90.5-100%, min - max). These results suggest that an average of 5 (4-19) locations within a station were needed to saturate diversity.

**
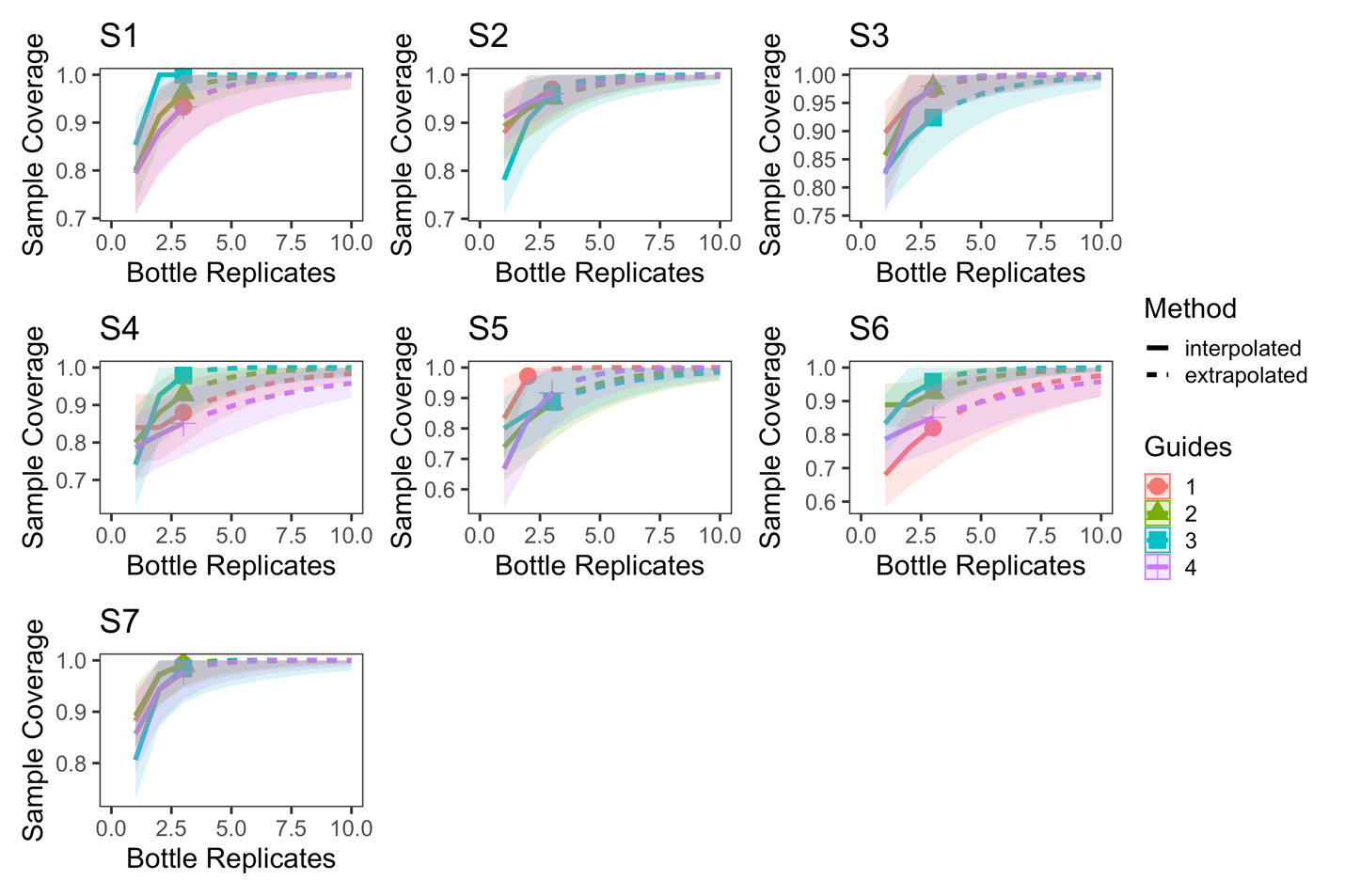
**

**Figure S4. eDNA Survey Location Level Sample Coverage Curves**

Sample coverage curves for eDNA surveys at the location level with each station plotted separately. The average sample coverage estimate across bottle replicates within each location was 94.0% (82.0%-100%, min - max).

**
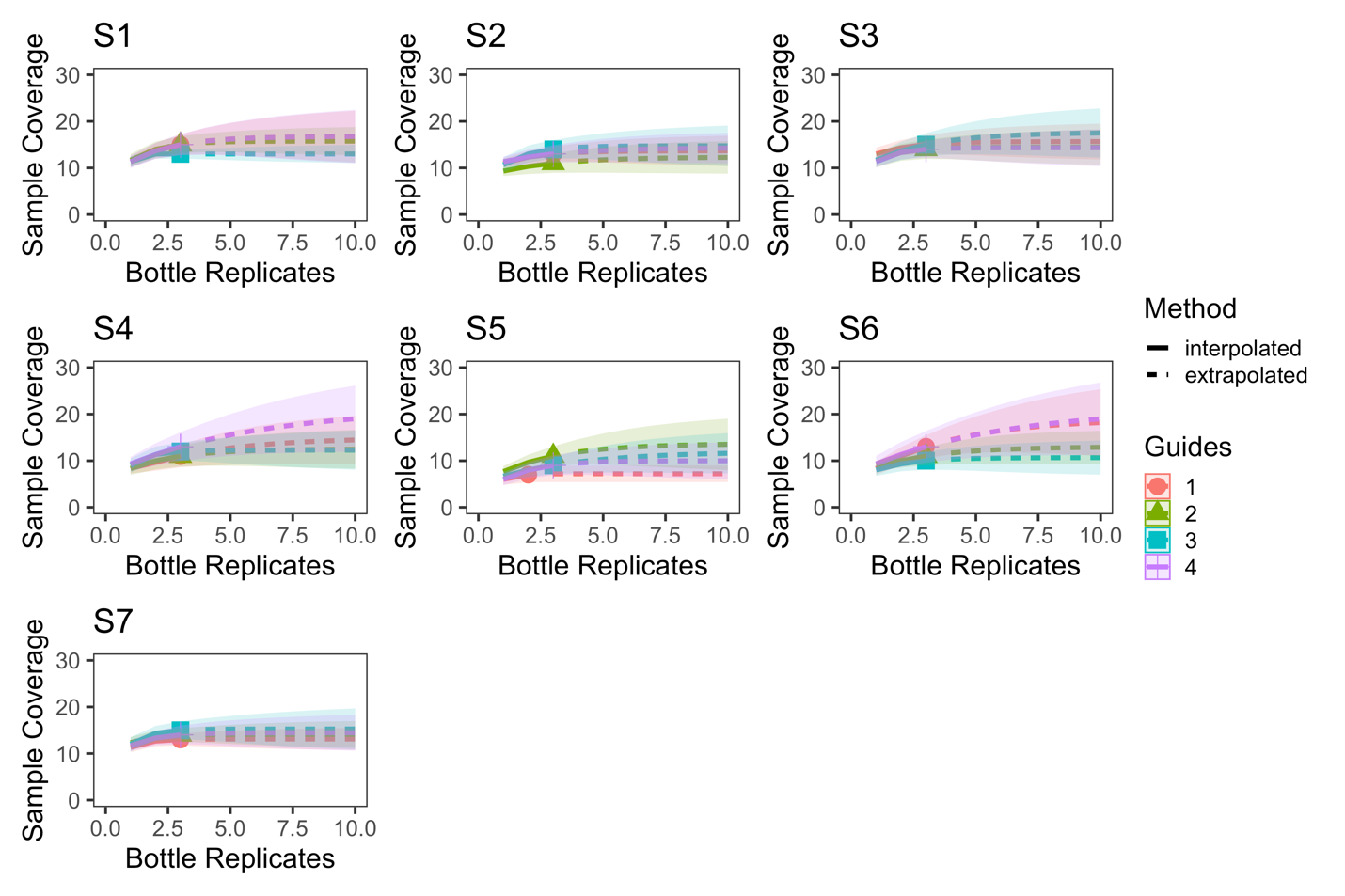
**

**Figure S5. eDNA Survey Location Level Species Rarefaction Curves**

Species rarefaction curves for eDNA surveys at the location level with stations plotted separately. These results suggest that an average of 7.3 (2-19) bottles within a location were needed to saturate diversity.

# S1. APPENDIX III: Biomass Comparisons

## Comparison of eDNA and Trawl Biomass Estimates

We compared eDNA index scores of relative abundance to trawl estimated biomass and fish counts for species detected at least three times (n=4) and for Northern Anchovy (*Engraulis mordax*) which was found in every eDNA sample. To investigate the relationship between eDNA index scores and trawl biomass, linear regressions between both metrics were fit in *R*, and *R*^2^ and equations for each fit are reported.

We found that eDNA index tracked biomass for three of these species [California lizardfish (*Synodus lucioceps*)*,* California halibut (*Paralichthys californicus*)*,* and Barred sand bass (*Paralabrax nebulifer*)] (R2>0.28), but did not track two species (White croaker *Genyonemus lineatus* and *E. mordax*) (Figures S6-7). The poor biomass correlation observed in *G. lineatus* was driven by one strong eDNA detection in which the trawl failed to catch any of the schooling White croaker. Likewise, eDNA detected *E. mordax* (also a schooling species) at every site, while trawls only collected 198 individuals at one site and 2 individuals at another. These results likely reflect the difficulty that a small number of trawls would have in sampling schooling (highly under-dispersed) species.


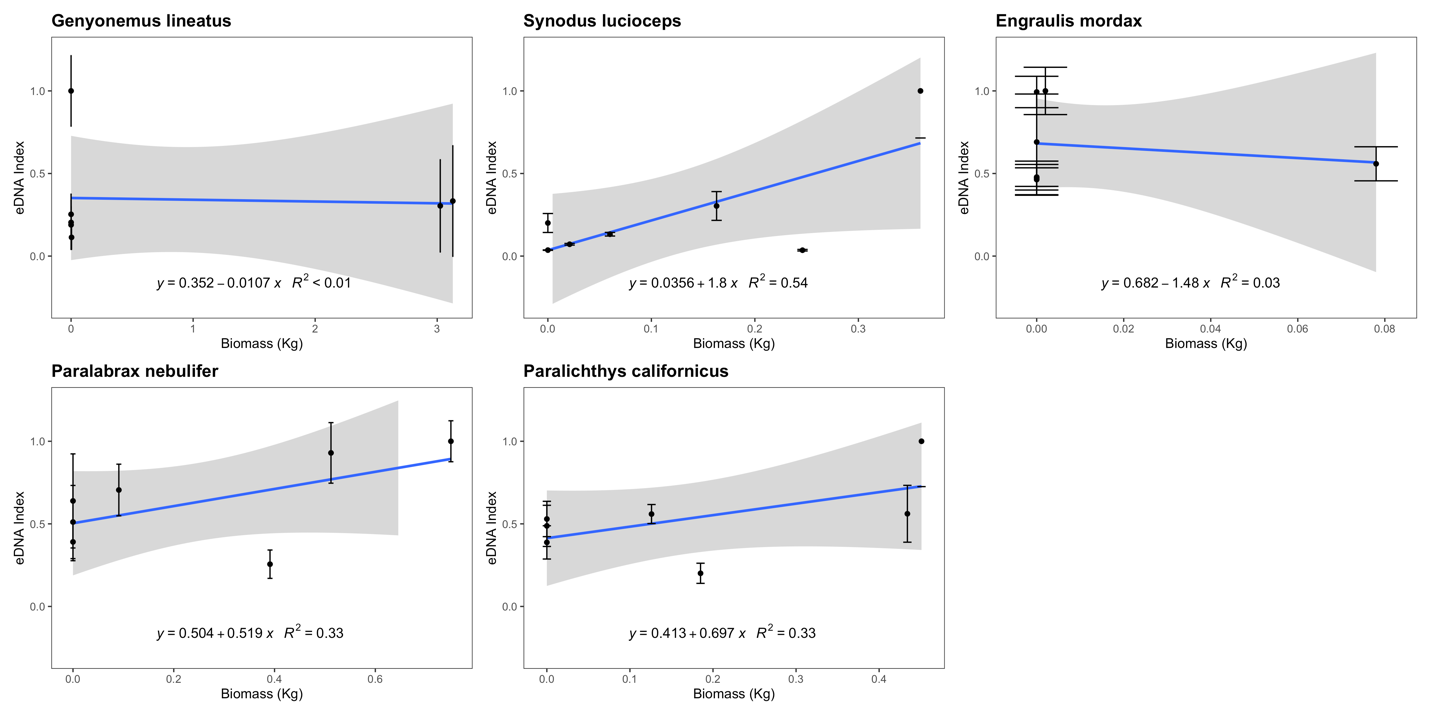


**Figure S6. eDNA Index vs. Trawl Biomass (kg)**

eDNA index tracked biomass for three of the five species with sufficient data points to be analyzed. We note that such correlations between eDNA metabarcoding results and visual observations are fraught with challenges as detailed in the Discussion section.

**
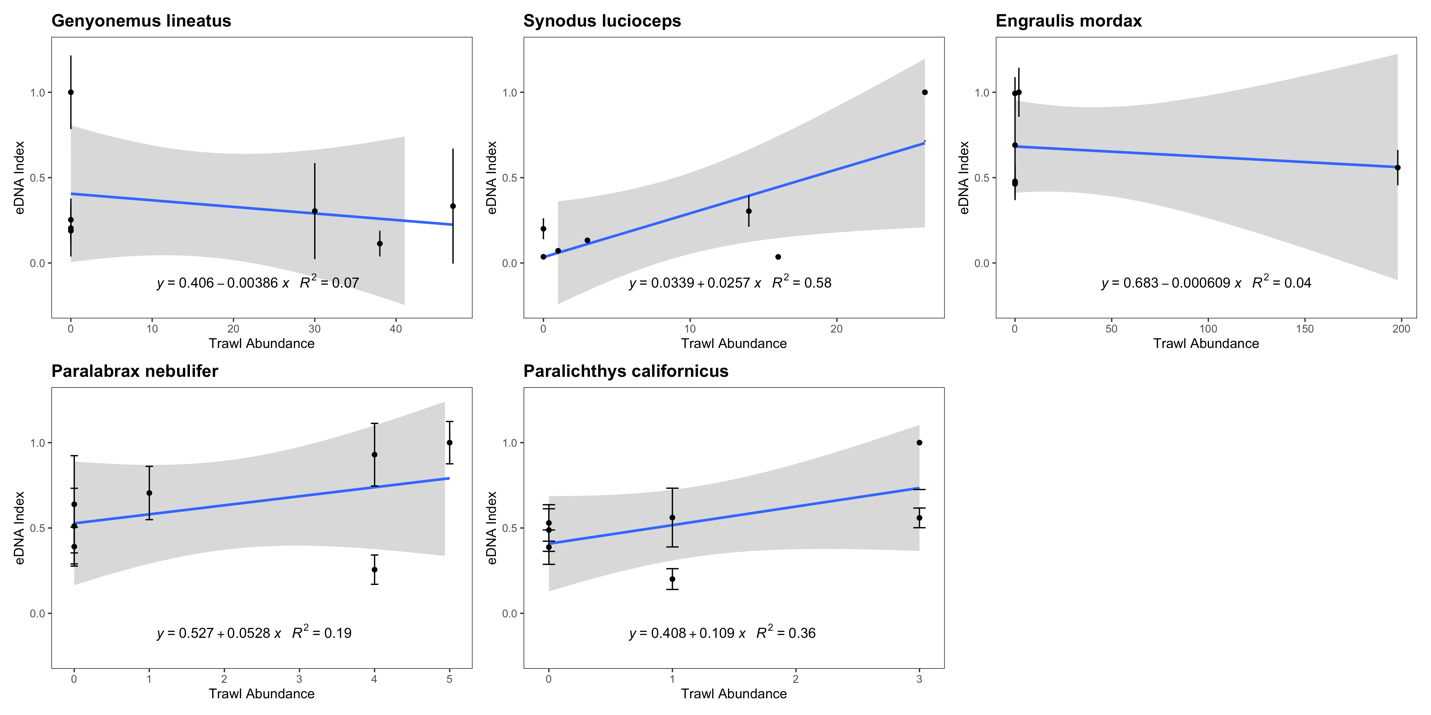
**

**Figure S7. eDNA Index vs. Trawl Abundance (counts)**

eDNA index tracked biomass for three of the five species with sufficient data points to be analyzed. We note that such correlations between eDNA metabarcoding results and visual observations are fraught with challenges as detailed in the Discussion section.

These results suggest an imperfect relationship between eDNA and trawl-derived estimates of biomass likely driven by the variability between repeated trawl tows (Shelton et al. 2019). In our case study, eDNA methods far more consistently detected species observed by trawls (Figure 5), suggesting that intra-site variability of eDNA methods may have been lower. However, correlations between methods conducted here only have seven corresponding data points, making any comparison limited at best.
